# Rotifers in space: transcriptomic response of the bdelloid rotifer *Adineta vaga* aboard the International Space Station

**DOI:** 10.1186/s12915-025-02272-1

**Published:** 2025-07-01

**Authors:** Victoria C. Moris, Lucie Bruneau, Jérémy Berthe, Richard Coos, Bjorn Baselet, Anne-Catherine Heuskin, Nicol Caplin, René Demets, Jutta Krause, Lobke Zuijderduijn, Alexandra Tortora, Magdalena Herova, Sébastien Penninckx, Luca Parmitano, Kevin Tabury, Sarah Baatout, Karine Van Doninck, Boris Hespeels

**Affiliations:** 1 Research Unit in Environmental and Evolutionary Biology (URBE), Institute of Life, Earth & Environment (ILEE), University of Namur, Namur, Belgium; 2 Laboratory of Molecular Biology & Evolution (MBE), Department of Biology, Université Libre de Bruxelles, Brussels, 1000 Belgium; 3 Laboratory of Analysis By Nuclear Reactions (LARN), Namur Research Institute for Life Sciences (NARILIS), University of Namur, Rue de Bruxelles, 61, Namur, B-5000 Belgium; 4 Radiobiology Unit, Belgian Nuclear Research Centre (SCK•CEN), Mol, Belgium; 5 ESTEC, Keplerlaan 1, Noordwijk, 2201 AZ Netherlands; 6 Kayser Italia S.R.L, Via Di Popogna, Leghorn, 501, 57128 Italy; 7 Lucerne School of Engineering and Architecture, BIOTESC, Hochschule Luzern Technik & Architektur, Obermattweg 9, Hergiswil, 6052 Switzerland; 8 Medical Physics Department, Institut Jules Bordet, Université Libre de Bruxelles, Brussels, Belgium; 9 EAC/JSC Liaison Officer, NASA’s Johnson Space Center, Houston, TX USA

**Keywords:** ISS, Space, Microgravity, Radiation, DNA repair, Bdelloid rotifers

## Abstract

**Background:**

The biological effects of spaceflight remain incompletely understood, even in humans (*Homo sapiens*), and are largely unexplored in non-traditional models such as bdelloid rotifers.

**Results:**

This study analyzes the transcriptomic changes experienced by *Adineta vaga*, a bdelloid rotifer aboard the International Space Station (ISS), using RNA sequencing. The aim was to investigate the overall effect of spaceflight in Low Earth Orbit (LEO) on these organisms. To this end, new hardware was developed to enable autonomous culturing of rotifers with minimal astronaut intervention. The study revealed significant transcriptomic changes, with 18.61% of genes showing differential expression in response to microgravity and radiation. These changes included upregulation of genes involved in protein synthesis, RNA metabolic processes, and DNA repair. Notably, the study also found a significant enrichment of foreign genes (Horizontal Gene Transfers: HGTs) among the genes that were either over- or under-expressed during spaceflight, suggesting that HGTs play a role in bdelloids’ adaptability to new and potentially atypical environments.

**Conclusions:**

This research not only enhances our understanding of how organisms respond to microgravity but also proposes *A. vaga* as a valuable model for future studies in space biology.

**Supplementary Information:**

The online version contains supplementary material available at 10.1186/s12915-025-02272-1.

## Background

As a result of millions of years of evolution on Earth’s surface, the human body has become intricately adapted to terrestrial conditions, leaving it unprepared for the unique challenges of the space environment [1]. In the space environment, humans are faced with two major biological hurdles, (1) coping with elevated levels of ionizing radiation, and (2) being confronted with the physiological and psychological ramifications of microgravity [2]. In Low Earth Orbit (LEO), where the International Space Station (ISS) is located, radiation levels reach about 0.3 Sv per year due to its orbital altitude and inclination. However, in deeper space regions beyond the cruising altitude of the ISS, the radiation doses are even higher, in stark contrast to the 0.005 Sv per year experienced on Earth, primarily from solar and cosmic radiation. Spaceflights are known to cause health hazards that impact a multitude of biological systems, creating among other oxidative stress, DNA damage, disruption of mitochondrial function, alterations in epigenetic and regulatory mechanisms, changes in telomere length, and imbalances in host-microbe interactions [3]. In addition to these, humans have to deal with confinement, isolation, the inherently hostile environment, and distance from Earth [4–6]. Today, further research is indispensable in order to better understand and monitor the impacts of spaceflights on living organisms. Enhancing our understanding of the space biological responses in biological model systems, including humans, will highlight pathways and mechanisms triggered by long-term spaceflights. In addition, the investigation of biological reactions that protect or repair the harmful consequences of space exposure will greatly contribute to the planning and execution of future space missions [3, 7].

To better understand space-induced phenomena, researchers have turned to various methodologies, with transcriptomics becoming an approach of choice. Using technologies such as microarray and RNAseq, transcriptomics enables a non-a priori approach into the impact of spaceflight on a broad range of biological pathways expressed in both spaceflight and ground reference samples. Historically, spaceflight research on the ISS has mainly focused on specific biological models (see Nasa Genelab database for example [8]), such as humans [7, 9], the mouse species *Mus musculus*, the worm *Caenorhabditis elegans* [10], and the fruit fly *Drosophila melanogaster* [11] for metazoans, and *Arabidopsis thaliana* for plants [12], along with several bacterial strains [13, 14]. Despite these significant efforts, the limited range of biological models has constrained our understanding and none of the studied metazoan species are radiation tolerant. We are still in the early stages of spaceflight-related biological research, and the data’s complexity makes a comprehensive view difficult, given that it can be influenced by many parameters. For example, various studies have aimed to explore bacterial transcriptomes’ response to spaceflight environments. These studies have been diverse in terms of organisms, media, culture conditions, and spaceflight hardware used, making cross-experiment analysis challenging. Meta-analyses highlighted that most of the variation seems to stem from differences in the experiments themselves rather than shared biological responses [13–16].

Bdelloid rotifers offer a unique opportunity to address some of these challenges due to their remarkable resilience and adaptability. Indeed, though minuscule and complex metazoans, bdelloid rotifers are notable for their remarkable resilience, adaptability, and unique reproductive strategy. Measuring less than 1 mm in length and comprising approximately 1000 cells, these eutelic animals (i.e., having a fixed number of cells at maturity) are equipped with fully developed nervous, muscular, digestive, and excretory systems [17]. One peculiar feature of bdelloid rotifers, being even more unusual among animals, is the complete absence of males or any male structures across the 460 described morphospecies [18]. These organisms are all-females, having propagated through parthenogenesis for millions of years [19]. Their asexual mode of reproduction has led to them being referred to as an “evolutionary scandal,” persisting without sexual reproduction and defying conventional evolutionary theories [20]. Predominantly inhabiting semi-terrestrial environments, bdelloid rotifers are found in diverse ecosystems across the globe. More than 90% of species are adapted to limno-terrestrial environments such as mosses and lichens [21]. This distribution is supported by their extraordinary tolerance to stresses like desiccation, freezing, deep vacuum, UV, and ionizing radiation [22–30].

The intriguing radiation resistance of bdelloid rotifers has been postulated to be an adaptation to survive desiccation, reflecting a high capacity to deal with numerous DNA double-strand breaks (DSBs) induced by both stresses [23, 30, 31]. Indeed, ionizing radiation (IR) inflicts cellular damage, notably through the formation of reactive oxygen species (ROS) and DNA DSBs [32]. Studies have shown that species like *Adineta vaga* can accumulate and efficiently repair DNA DSBs even after severe proton, iron, or X-ray irradiation, and following complete desiccation [25, 26]. Further investigations by Krisko et al. revealed that *A. vaga* is significantly more resistant to IR-induced protein carbonylation compared to the more radiosensitive nematode *C. elegans* [33]. This echoes the resilience found in the radiation-resistant bacterium *Deinococcus radiodurans* [34], suggesting that bdelloid rotifers survive extreme conditions through a combination of efficient antioxidants and DNA repair mechanisms [30, 33, 35–37].

In pursuit of understanding life’s adaptability to space, we propose the use of bdelloid rotifers, specifically the model species *A. vaga* whose genome, transcriptome, and proteome are available, as pioneering model species for space research. This species presents an array of unique characteristics that make them particularly suited for this role (adapted from [38]). (1) Miniaturization: Ranging from 150 µm to 1 mm, bdelloid rotifers’ small size allows for the conduct of experiments with multiple individuals in constrained spaces, a vital consideration for space-based research. (2) Complexity: Despite their minuscule size, bdelloids possess a remarkable level of biological intricacy, featuring multicellular organization, tissues, organs, a complete gut, and complex muscles. This complexity enables the study of various biological phenomena within a small organism. (3) Availability: Bdelloid rotifers are both prevalent in nature and amenable to cultivation under controlled conditions, facilitating accessibility for experimental use. (4) Short Life Span: With brief life cycles, bdelloid rotifers can be studied over multiple generations within a reasonable time frame, accelerating research progress. (5) Asexual Reproduction: The absence of males in bdelloid populations enables rapid propagation of cultures with reduced genetic variability. A single individual can quickly produce a genetically cohesive population, offering opportunities for consistent experimentation. (6) Extremotolerance: Known for their ability to withstand high levels of DNA damage and various stressors likely to be encountered during space flight, bdelloids are invaluable for studying mechanisms of extremotolerance, which can shed light on the adaptation of life in space. (7) Storage: Bdelloid rotifers’ unique ability to survive desiccation and freezing allows for convenient storage before and after scientific experiments, being able to resurrect dried or frozen experimental clones. This preservation has minimal impact on their biology, ensuring consistent and reliable experimental results. (8) Desiccation Resistance: The inherent desiccation resistance of bdelloid rotifers correlates with increased resilience to extreme temperatures and the vacuum of deep space. This makes them ideal candidates for exploring the boundaries of life in space, particularly during exobiology experiments. Leveraging the unique advantages of bdelloid rotifers—such as their growth in constrained experimental setups and their representative multicellular complexity—offers a powerful model for studying how key genetic pathways are modulated under spaceflight conditions. For example, conserved pathways involved in DNA replication/repair, protein folding, and oxidative stress management are essential for the health and function of higher organisms, including humans. By investigating these processes in bdelloids, we expect to provide valuable insights that can help guide the development of strategies to counteract the adverse effects of space stressors in more complex systems. Moreover, the remarkable resilience of bdelloid rotifers to extreme conditions enables the discovery of novel stress resistance mechanisms with broad implications for understanding cellular adaptation in space, ultimately enhancing both experimental robustness and translational relevance.

In this study, we specifically aim to evaluate the effect of microgravity on the biological processes of the bdelloid rotifer species *A. vaga* through a large-scale transcriptome analysis (RNAseq) to screen pathways that could be affected by the space flight environments in LEO. In close collaboration with the European Space Agency (ESA), an experimental design was implemented to address the impact of spaceflight on hydrated bdelloid rotifers. Autonomous cultures of *A. vaga* individuals were transported to the ISS and cultured for 7 days onboard of the ISS at a constant temperature (15 °C). After 1 week, the growth was stopped by fast freezing using − 80 °C MELFI freezer on board ISS. Parallel ground control samples were provided for proper comparison. This study is the first step of several experiments using *Adineta vaga* as biological model system to characterize the biological response of complex metazoans evolving in space environment. These experiments contribute, in combination with the multitude of data collected from other ground-based and space investigations, to face the biological challenges occurring during space exploration. Furthermore, genetic and molecular analyses of rotifers can enhance our knowledge of biological resilience, informing future space missions and astrobiology research.

## Results

### Successful exposure of hydrated A. vaga individuals in the ISS’s KUBIK facility and ground control experiments

**Fig. 1:**
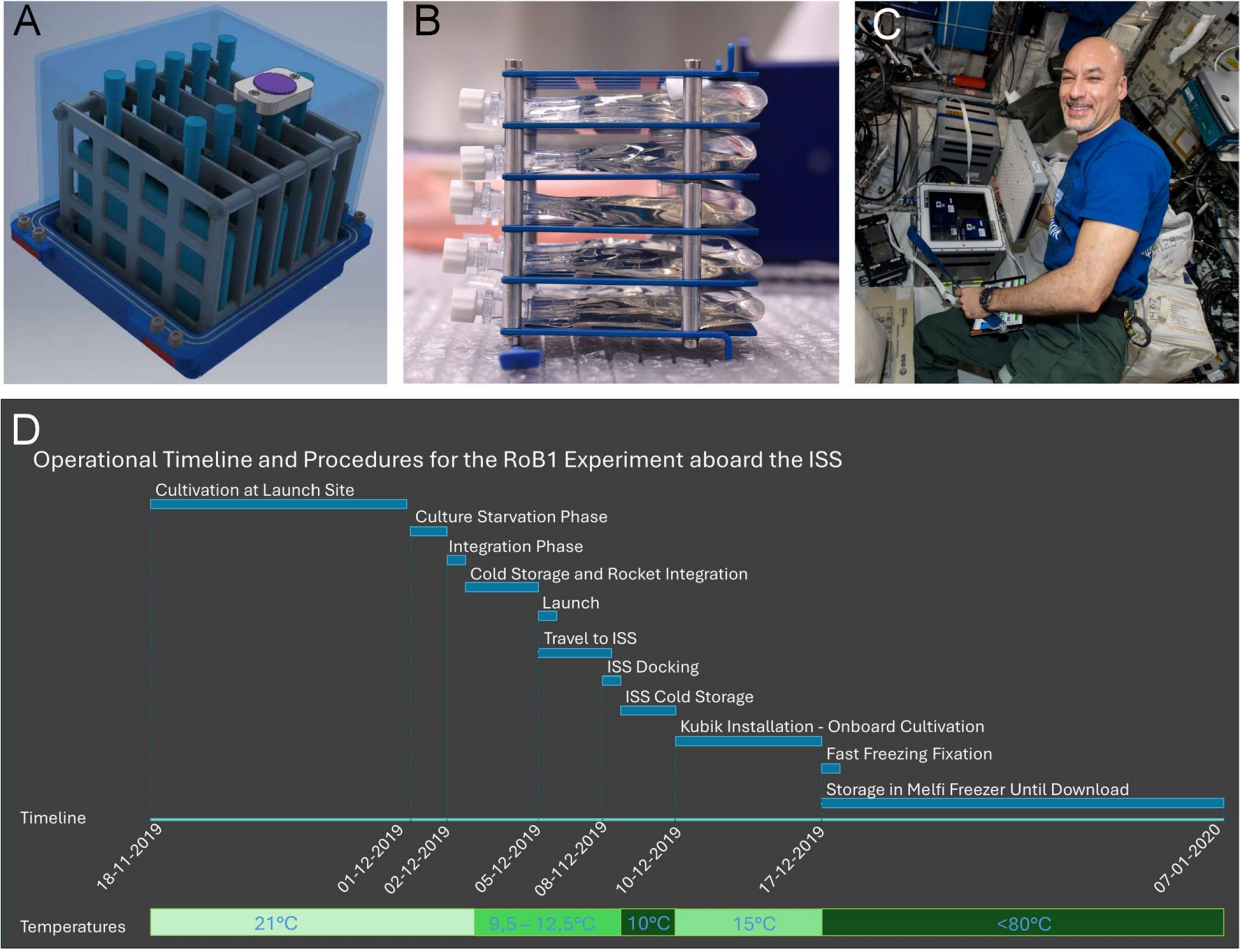
**A** Assembled Hardware with a transparent view illustrating the internal hardware frame and culture bag. On the top, iButton is visualized as purple ring. Photo credits: Kayser Italia **B**) View of the culture bag loaded inside the internal frame of hardware. Each bag contains 10,000 *A. vaga* individuals. Photo credits: Marc Guillaume. **C** ESA astronaut Luca Parmitano handling the experiment on board the ISS. Photo credits: ESA. **D** Operational Timeline and Procedures for the experiment. Similar timeline and temperature were achieved for space and ground samples

*A. vaga* individuals were cultured in specially designed hardware (Fig. 1A, B) onboard the ISS from December 10 th to December 17 th, 2019, using the KUBIK incubator (Fig. 1C) maintained at a temperature of 15 °C (Fig. 1D). Onboard the ISS (i.e., from docking to fixation), radiation exposure was monitored as part of the permanent DOSIS 3D radiation system. The total absorbed dose in H₂O was 3.94 mGy, and the total dose equivalent was 8.49 mSv, as measured by the DOSTEL-2 instrument, which is part of the DOSIS 3D system, inside the Columbus Laboratory of the ISS. Throughout the various stages of the experiment—encompassing upload, cold storage, cultivation within KUBIK, freezing and cold storage at − 80 °C, and download till laboratory recovery—all operations proceeded without any technical complications. As reference, ground control samples were maintained with parameters closely mirroring those of the flight samples, except for the absence of microgravity and the natural radiation profile on board ISS.

Screening of visual controls revealed that the rotifers remained active throughout the entire experiment (*Additional file 1: Fig. S1*, *Additional file 2: movie1* showing an overview of ground control *A. vaga* individuals in a PL30 before fixation). Before fixation of control samples, we identified the presence of numerous eggs (*Additional file 1: Fig. S2* and *Additional file 2*), an indication that the bdelloid rotifers were reproductively active. This finding was further corroborated by the observed increase in specimen density as the experiment progressed. Equally notable was the absence of any unanticipated fungi or bacteria populations, which could have potentially clouded the cultivation medium, thereby imposing stress on the cultivated specimens. Presence of egg clusters was also reported in flight samples, after return to Earth, supporting the effective reproduction of animals in space.

Post-experiment hardware disassembly enabled RNA extraction, yielding a minimum of 510 ng pure RNA per sample. Out of these, 8 of the 10 flight samples and an equivalent number of ground samples were earmarked for RNA sequencing.

### Differential gene expression between flight and ground samples

**Fig. 2:**
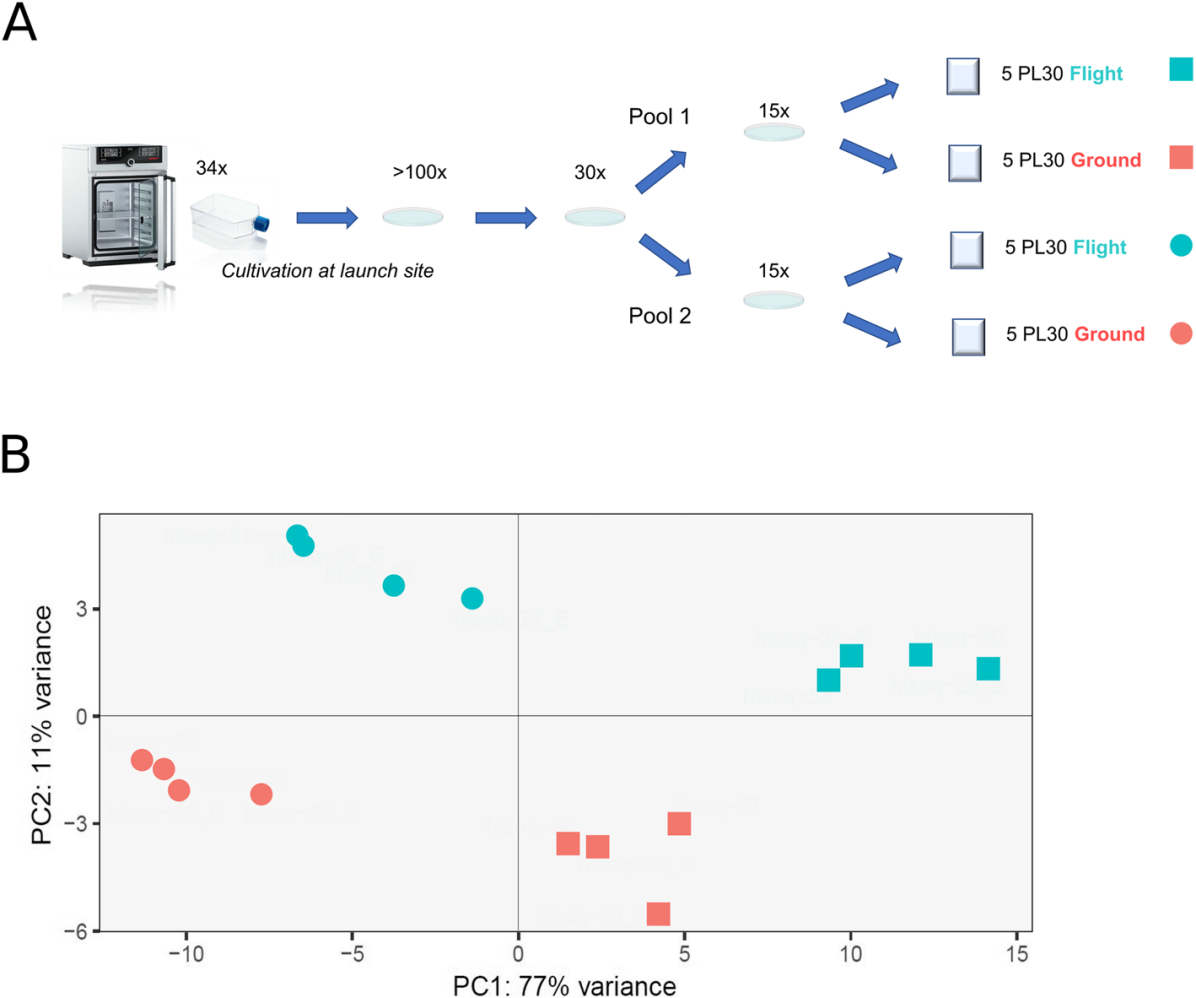
**A** Experimental design of Rob1 experiment; **B** principal component analysis (PCA) of 16 transcriptomes (8 ground samples: 4 Pool 1, 4 Pool 2; 8 flight samples: 4 Pool 1, 4 Pool 2)

After sequencing, 9–44 million reads were obtained per sample (*Additional file 3: Table S1*). Trimming the adapters resulted in a removal of a maximum of 1.3% of the raw reads. Between 85 and 89% of these trimmed reads were uniquely mapped (*Additional file 3: Table S1*) on the *A. vaga* genome. Statistically significant differentially expressed genes were identified in the transcriptomes of *A. vaga* under two conditions: onboard the ISS (hereafter referred to as “flight,” *N* = 8) and on Earth (hereafter referred to as'ground', *N* = 8). For each condition, four samples from two distinct pools (Pool 1 and Pool 2) were utilized (*Additional file 3: Table S1*; Fig. 2A). The bioinformatic assessment revealed notable biological variation among rotifers sourced from the two biological material sets. A significant effect attributable to the genetic pools was evident (Fig. 2B), accounting for 77% of the variance. This pronounced biological variability was unanticipated given the asexual mode of reproduction, although with meiotic recombination possible [39]. Yet, the presence of multiple replicates (8 Flight and 8 Ground) mitigated this variation effect.

**Fig. 3:**
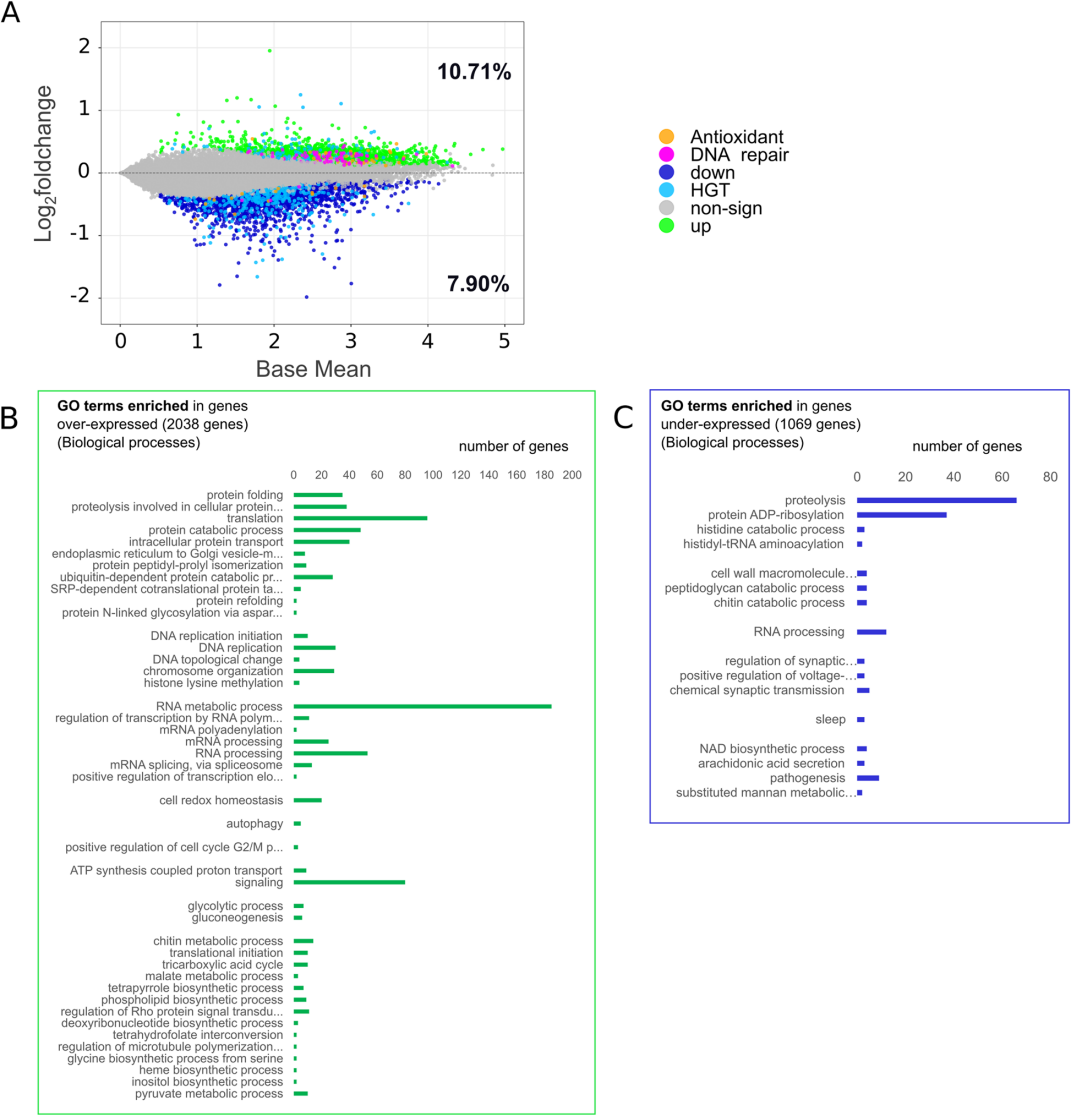
**A** Differential plot showing significantly differentially expressed genes (colored dots, other than gray) over-expressed (log2foldchange > 0 in green) and under-expressed (log2foldchange < 0 in dark blue) in *A. vaga* specimens under flight condition compared to those under the ground condition. Genes differentially expressed and involved in DNA repair are colored in magenta, those coding for antioxidants in orange and identified as HGTs in light blue. *Y* axis represents the log2foldchange and *x* axis a transformation of the mean expression of the genes (see calculation in Methods). **B** Significant GO Biological processes (BP) enriched in over-expressed genes; the *x*-axis shows the number of over-expressed genes characterized for a specific GO BP (*y* axis). **C** Significant GO Biological processes enriched in under-expressed genes; the *x*-axis shows the number of under-expressed genes characterized for a specific GO BP (*y* axis)

To decipher the differential gene expression between the two environments (flight vs. ground), the genetic variability was treated as a batch effect, and analysis was conducted using DESeq2 software. A notable effect on gene expression due to the given condition was also apparent, accounting for 11% of the variance (Fig. 2B). Taking into account the genetic pool, there were 3153 genes over-expressed (log2foldchange > 0, FDR < 0.05; representing 10.71% of the entire genome) and 2327 genes under-expressed (log2foldchange < 0, FDR < 0.05; representing 7.90% of the entire genome) in *A. vaga* individuals of the flight environment as compared to those in the ground setting (Fig. 3A*, Additional file 1: Fig. S4*). However, the majority of these genes exhibited positive or negative log2foldchange gene expression differences ranging from 0 to 0.5. In fact, merely 82 genes exhibited a log2foldchange > 0.5, while 596 demonstrated a log2foldchange < − 0.5. Similar results were found with EdgeR (*Additional file 1: Fig. S3*, *Additional file 3: Table S2*), with mostly genes with low log2foldchange being sometimes not supported by the two approaches.

### Gene Ontology enrichment analyses

To delineate the biological processes influenced by spaceflight, an enrichment analysis of differentially expressed (DE) genes was undertaken using Gene Ontology (GO). While a significant number of genes were not associated with any specific GO term (1115 over-expressed genes and 1258 under-expressed genes; *Additional file 1: Fig. S5*), an enrichment analysis was executed for genes that had corresponding GO terms (2038 over-expressed and 1069 under-expressed genes; Fig. 3A).

Several enriched biological processes were found within the genes over-expressed in *A. vaga* specimens that were on the ISS compared to those which stayed on Earth (Fig. 3B). Notably, these processes were involved in protein modifications, including refolding, proteolysis, and translation, alongside DNA replication, topological changes, RNA metabolic processes, and other metabolic pathways.

For genes that were under-expressed in the flight *A. vaga* individuals, enriched processes were mainly centered on protein modifications, notably proteolysis, ADP-ribosylation, and histidine processes (Fig. 3C). Additionally, these genes were integral to RNA processing, peptidoglycan/chitin catabolic pathways, synaptic modulation, and transmission, etc. (Fig. 3C).

### Analysis of top ranked genes and their specific functions

**Fig. 4:**
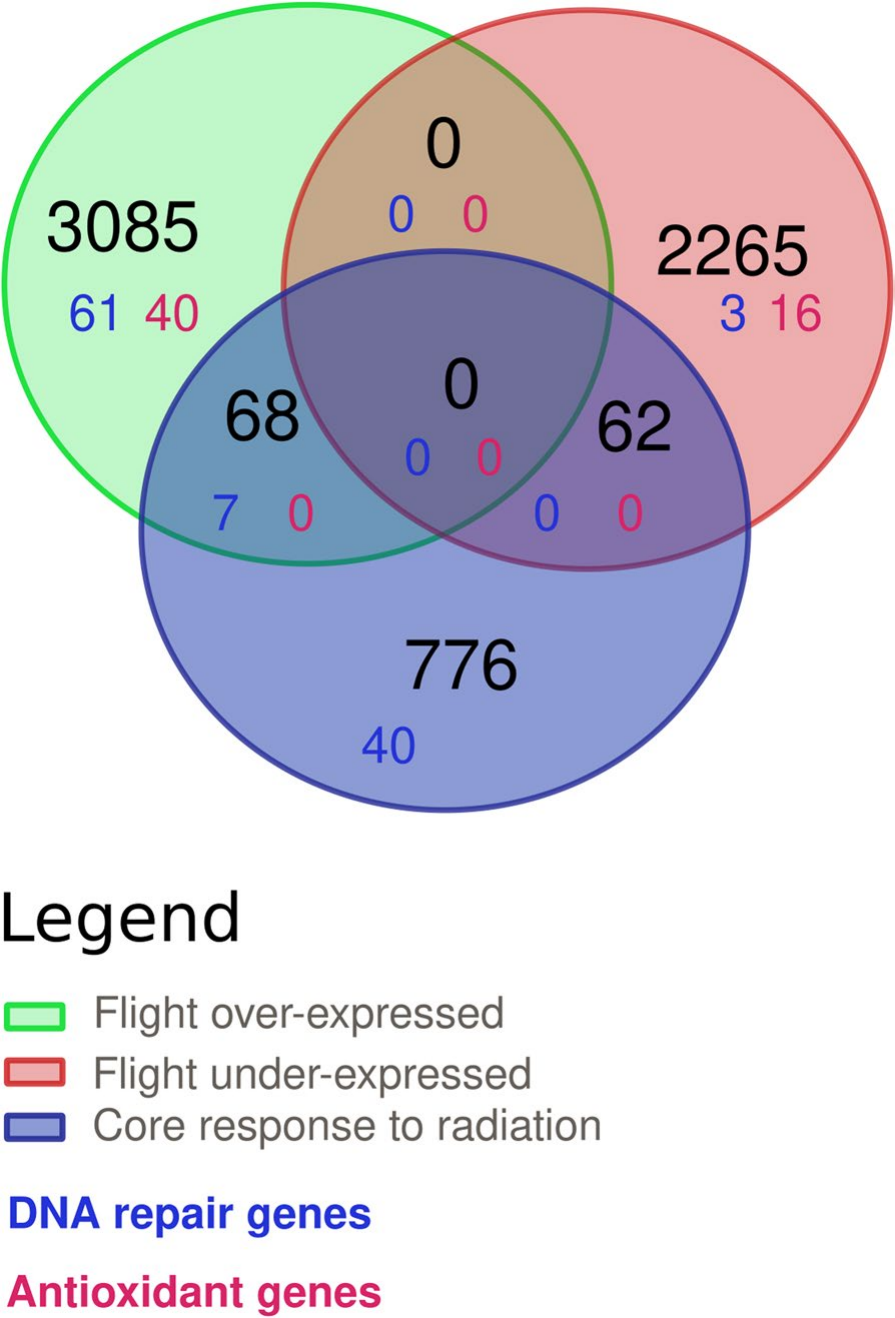
Venn diagrams of genes over- (light green) and under- (light red) expressed in *A. vaga* specimens under the flight condition compared to those under the ground condition with over-expressed genes identified as a core response to different kinds of radiation (X-rays, Fe ions) in desiccated or hydrated state (dark blue)

Of the 3153 genes over-expressed during spaceflight, we closely examined the 25 genes that exhibited the highest log2foldchange values, ranging from 1.95 to 0.66 (Table 1, Additional file 1: Fig. S6). The function of 14 of these genes remains undefined as they lacked characterization via BLAST, Gene Ontology (GO), or Pfam terms. Ten genes demonstrated hypothetical functions or were associated to certain biological processes inferred from GO terms and BLAST results. These are thought to be involved in mannan metabolic processes (FUN_007147-T1, FUN_007125-T1), sugar hydrolysis (FUN_020880-T1), phosphoenolpyruvate carboxykinase (GTP) activity (FUN_027063-T1), ABC transporter functions (FUN_024281-T1), isocitrate lyase activity (FUN_023798-T1), and proteasome activator complex subunit 3 functions (FUN_023905-T1). The gene FUN_010444-T1 was only characterized by a characteristic repeat domain. Another gene, FUN_001315-T1, was primarily described by its GO annotation, associating it with membrane processes, while FUN_027063-T1 was associated with phosphoenolpyruvate carboxykinase (GTP) activity based on both GO and PFAM annotations. In total, six of the most differentially expressed genes during spaceflight were identified as HGT through Alienomics, including FUN_010444-T1, FUN_007125-T1, FUN_023905-T1, FUN_014542-T1, FUN_028525-T1, and FUN_020880-T1. Finally, two genes (FUN_027504-T1; FUN_028525), with unknown functions, were found in the core response to radiation ([30]; Table 1, Fig. 4).

**Table 1:** 25 top ranked genes with highest log2foldchange values over-expressed under the flight condition. The columns detail the gene ID, if they are identified as HGT, if the genes were also over-expressed in the core radiation response [30], the predicted function based on blast search, the species and evalue of the search, the GO ID, the Pfam domain identified, the log2foldchange (l2fc), and the false discovery rate (FDR)

Gene_id	HGT	Core	Predicted function	Evalue	Species	GO	Pfam	l2fc	FDR
FUN_001512-T1								1.95	6.31E − 38
FUN_007147-T1	HGT		Mannan endo-1,4-beta-mannosidase A and B	7.0e − 121	*Paenibacillus mucilaginosus*	GO:0006080	PF02156,Glycosyl hydrolase family 26	1.25	2.25E − 35
FUN_010499-T1								1.20	8.63E − 13
FUN_003516-T1								1.17	6.79E − 20
FUN_023399-T1								1.16	7.59E − 13
FUN_010444-T1	HGT		WP_018619370.1 hypothetical protein	1.9e − 42	*Spirosoma luteum*		PF13517,Repeat domain in Vibrio, Colwellia, Bradyrhizobium and Shewanella	1.11	1.59E − 21
FUN_013146-T1								1.07	3.91E − 10
FUN_007125-T1	HGT		Mannan endo-1,4-beta-mannosidase A and B	6.8e − 26	*Paenibacillus mucilaginosus*			1.05	6.85E − 14
FUN_023905-T1	HGT		Proteasome activator complex subunit 3	4.7e − 86	*Hondaea fermentalgiana*			1.05	2.42E − 17
FUN_026312-T1^a^								0.93	3.56E − 08
FUN_001315-T1						GO:0016020		0.87	8.91E − 09
FUN_011231-T1								0.82	2.11E − 07
FUN_013252-T1								0.82	3.87E − 06
FUN_023798-T1*			Isocitrate lyase	1.1e − 115	*Tepidicaulis*	GO:0004451	PF00463,Isocitrate lyase family	0.81	6.44E − 06
FUN_024281-T1			XP_001693039.1 predicted protein	8.0e − 194	*Chlamydomonas reinhardtii*	GO:0016020	PF00005,ABC transporter	0.81	7.14E − 15
FUN_010324-T1								0.80	2.96E − 20
FUN_027063-T1						GO:0004613	PF00821,Phosphoenolpyruvate carboxykinase C-terminal P-loop domain	0.76	6.25E − 23
FUN_000645-T1								0.74	4.08E − 05
FUN_015485-T1								0.73	4.75E − 13
FUN_013189-T1								0.71	2.06E − 08
FUN_014542-T1	HGT		WP_052672505.1 hypothetical protein	3.4e − 68	*Aliterella atlantica*	GO:0016021		0.71	3.11E − 10
FUN_027504-T1		X						0.71	1.69E − 08
FUN_028525-T1^a^	HGT	X	|XP_003287969.1 hypothetical protein DICPUDRAFT_33321	5.8e − 33	*Dictyostelium purpureum*			0.71	0.0001
FUN_004023-T1								0.69	2.96E − 06
FUN_020880-T1	HGT		Glycoside hydrolase 45	7.5e − 156	*Adineta ricciae*	GO:0005576	PF00734,Fungal cellulose binding domain	0.66	1.03E − 12

^a^Genes not validated by EdgeR

Within the 2327 genes under-expressed in specimens on the ISS relative to those on Earth, we focused on the 25 with the lowest log2fold change values, spanning from − 1.98 to − 1.13 (Table 2*, Additional file 1: Fig. S7*). As with the over-expressed genes, deducing the function or associated biological processes for these 25 genes proved challenging. Only 16 of these genes could be annotated using BLAST, GO, or Pfam, with five identified as HGTs. Based on these annotations, these genes are coding for proteins predicted to be astacin (peptidase family: FUN_014298-T1), human growth factor (FUN_023468-T1), 50S ribosome-binding GTPase (FUN_023706-T1), monocarboxylate transporter (FUN_014986-T1), retinol binding protein receptor (FUN_025721-T1), dockerin (FUN_000351-T1), and histidine ammonia-lyase-like (FUN_014988-T1) (Table 2).

**Table 2:** Twenty-five top ranked genes with highest log2foldchange values under-expressed under the flight condition. The columns detail the gene ID, if they are identified as HGT, the predicted function based on blast search, the species and evalue of the search, the GO ID, the Pfam domain identified, the log2foldchange (l2fc), and the false discovery rate (FDR). None of these genes were over-expressed in the core radiation response

Gene_id	HGT	Predicted function	evalue	species	GO	Pfam	l2fc	FDR
FUN_014647-T1							−1.98	1.34E − 57
FUN_014298-T1		Pentapeptide repeat family protein	4.6e − 37	*Adineta vaga*	GO:0006508^b^	PF01400,Astacin (Peptidase family M12 A)	−1.79	2.43E − 28
FUN_017589-T1							−1.77	1.90E − 77
FUN_023845-T1	HGT				GO:0005506		−1.66	1.39E − 27
FUN_000759-T1							−1.65	4.54E − 26
FUN_023468-T1		Uncharacterized protein LOC107451935	1.9e − 23	*Parasteatoda tepidariorum*	GO:0005509	PF12661,Human growth factor-like EGF	−1.51	1.16E − 43
FUN_001043-T1		Uncharacterized protein LOC111125451 isoform X2	4.5e − −59	*Crassostrea virginica*			−1.44	2.57E − 19
FUN_010437-T1	HGT						−1.43	3.64E − 23
FUN_014775-T1	HGT	T9SS C-terminal target domain-containing protein. partial	1.6e − 10	*Pontibacter roseus*			−1.39	3.50E − 24
FUN_015336-T1							−1.39	2.62E − 35
FUN_023706-T1	HGT	50S ribosome-binding GTPase	8.0e − 65	*Lyngbya aestuarii*		PF00350,Dynamin family	−1.38	2.05E − 22
FUN_014986-T1		Monocarboxylate transporter 14 isoform X1	2.6e − 20	*Lingula anatina*	GO:0055085	PF07690,Major Facilitator Superfamily	−1.37	2.35E − 43
FUN_023412-T1		Sushi domain-containing 2-like	5.6e − 87	*Brachionus plicatilis*	GO:0007160	PF03782,AMOP domain	−1.37	5.25E − 18
FUN_012134-T1							−1.32	8.43E − 28
FUN_000351-T1	HGT	dockerin	6.0e − 105	*Paenibacillus kribbensis*			−1.30	2.51E − 47
FUN_025721-T1		Stimulated by retinoic acid 6-like protein	4.0e − 139	*Adineta vaga*		PF14752,Retinol binding protein receptor	−1.28	3.35E − 14
FUN_009754-T1		VCBS repeat-containing protein	2.1e − 06	*Donghicola tyrosinivorans*			−1.25	5.26E − 23
FUN_028517-T1							−1.24	5.82E − 33
FUN_000584-T1							−1.23	1.34E − 23
FUN_001089-T1		Uncharacterized protein LOC111125451 isoform X2	4.4e − 59	*Crassostrea virginica*			−1.21	3.68E − 15
FUN_014988-T1		Histidine ammonia-lyase-like isoform X1	2.2e − 190	*Branchiostoma belcheri*	GO:0005737	PF00221,Aromatic amino acid lyase	−1.19	1.39E − 27
FUN_009425-T1^a^							−1.15	1.99E − 11
FUN_014858-T1^a^						PF11630,Protein of unknown function (DUF3254)	−1.14	3.01E − 11
FUN_024542-T1		Hypothetical protein RvY_16559	1.9e − 10	*Ramazzottius varieornatus*	GO:0003824		−1.14	4.83E − 12
FUN_013766-T1		Hypothetical protein B7P43_G04997	1.4e − 17	*Cryptotermes secundus*		PF16013,Domain of unknown function (DUF4781)	−1.14	9.31E − 32

^a^Genes not validated by EdgeR

^b^GO:0006508 enriched: proteolysis

Delving deeper into the dataset, according to annotations from Moris et al. [30] based on KEGG, pfam domains, and best blast hits, 68 genes over-expressed (log2foldchange > 0) in the space environment were linked to DNA replication and DNA repair pathways, spanning mechanisms such as Nucleotide Excision Repair (NER), Base Excision Repair (BER), Homologous Recombination (HR), Non-Homologous End Joining (NHEJ), and Mismatch Repair (MR) (Table 3*, Additional file 1: Fig. S8*). Those with the highest log2foldchange (L2fc > 0.33) were coding for proteins involved in replication (MCM4, MCM6, MCM7), in NER (TFIIH3), in HR (BRAC1), in NHEJ (Artemis), or in cross pathways (PCNA, RCF1, polymerase epsilon, RFA). Among these genes, those with the highest expression level (TPMs) were coding for high mobility proteins, likely involved in BER (FUN_022518-T1, FUN_006214-T1). Only three genes involved in DNA repair were under-expressed: two von willebrandt factor type a domain containing protein (FUN_009967-T1, FUN_010278-T1), and one gene coding for a PARP (FUN_011186-T1) [30]. On contrary to radiation response, we observed more genes involved in DNA replication and HR to be over-expressed (e.g., Rad50, Rad51). Indeed, in accordance with the GO significant enrichment, many genes involved in DNA replication were over-expressed in the Flight condition (Table 3).

**Table 3:** Genes predicted to code for proteins involved in DNA repair being over-expressed (Log2foldchange: L2fc > 0, FDR < 0.05) in Flight condition compared to Ground condition. The columns detail the gene ID, if they are identified as HGT, if the genes were also over-expressed in the core radiation response [30], the pathway in which the protein is involved, the protein name, the log2foldchange, FDR, and Transcripts per Millions (TPMs) in Ground (G) and in the Flight (F) conditions for the two pools : Team 1 (T1) and Team 2 (T2)

Gene ID	HGT	Core	Pathway	Protein	L2fc	FDR	G_T2	G_T1	F_T2	F_T1
FUN_012833-T1			REP	MCM4, CDC54	0,44	0,00	14,72	27.71	19.07	37.67
FUN_002254-T1			REP	MCM7, CDC47	0.41	0.00	17.34	32.20	21.73	42.41
FUN_021625-T1			NER	TFIIH3, GTF2H3, TFB4	0.37	0.00	9.24	11.69	11.02	14.93
FUN_025314-T1			HR	BRCA1	0.35	0.02	1.33	1.85	2.09	1.89
FUN_012831-T1			cross	PCNA	0.34	0.00	14.25	22.36	16.41	29.06
FUN_008446-T1			cross	RFC1	0.33	0.00	8.09	11.30	10.30	13.01
FUN_027891-T1			cross	POLE2	0.33	0.00	12.78	16.27	15.46	19.55
FUN_002012-T1			cross	RFA/RPA	0.33	0.01	43.94	79.04	55.03	95.97
FUN_006663-T1		X	NHEJ	Artemis	0.33	0.02	2.51	2.18	3.58	2.54
FUN_003209-T1			REP	MCM6	0.32	0.00	17.06	28.39	19.65	35.81
FUN_026481-T1			NER	XPC	0.32	0.00	6.54	7.96	7.85	9.66
FUN_028066-T1			HR	XRCC3	0.32	0.00	5.15	6.60	6.26	8.22
FUN_018514-T1			HR	BARD1	0.32	0.00	8.89	14.58	10.82	17.55
FUN_025542-T1			HR	TOP3	0.32	0.00	19.52	21.49	25.11	24.42
FUN_019127-T1			REP	MCM7, CDC47	0.31	0.00	27.83	38.64	31.50	48.46
FUN_025441-T1			REP	MCM2	0.31	0.01	26.22	39.21	31.02	47.67
FUN_002957-T1			BER	UNG, UDG	0.31	0.00	47.44	65.88	56.12	78.53
FUN_018804-T1			NER	RPB6	0.31	0.01	45.37	63.74	52.24	80.12
FUN_026163-T1		X	NHEJ	APLF	0.30	0.03	6.97	8.99	8.10	10.68
FUN_003120-T1			NER	cyclin-dep. kinase 7	0.30	0.00	48.52	68.66	57.86	79.88
FUN_022518-T1			BER	high mob	0.30	0.02	912.04	1443.67	1095.76	1722.63
FUN_026614-T1			REP	MCM5, CDC46	0.29	0.01	38.24	52.68	43.45	64.18
FUN_025305-T1			HR	BLM, RECQL3, SGS1	0.29	0.01	7.91	9.26	9.34	10.85
FUN_026326-T1			BER	APEX1	0.29	0.02	37.55	58.58	43.66	70.35
FUN_022601-T1			HR	RAD51	0.28	0.01	16.60	30.88	20.33	35.02
FUN_015896-T1			REP	PRI1	0.28	0.00	10.79	15.92	12.01	19.44
FUN_007194-T1			HR	RAD54L, RAD54	0.28	0.00	13.07	21.43	15.87	23.85
FUN_025614-T1			REP	MCM3	0.27	0.01	41.43	55.08	47.49	64.84
FUN_020478-T1			NER	ERCC2, XPD	0.27	0.00	8.70	10.87	10.74	12.15
FUN_004147-T1			NER	RAD23, HR23	0.27	0.00	141.65	156.74	159.14	185.17
FUN_006758-T1			BER	mutM, fpg	0.27	0.00	36.35	47.39	40.91	56.02
FUN_018150-T1			cross	RFC3_5	0.27	0.00	30.59	39.09	34.53	46.42
FUN_008727-T1			cross	RFC2_4	0.27	0.00	48.10	52.85	54.99	61.76
FUN_027556-T1		X	cross	PCNA	0.27	0.01	90.46	126.54	101.83	151.72
FUN_017933-T1			Direct Rev	PHRB	0.26	0.03	9.39	12.49	10.84	14.68
FUN_021025-T1			cross	RFC3_5	0.26	0.03	24.82	34.37	26.97	42.77
FUN_005925-T1			MR	EXO1	0.25	0.03	8.22	12.17	9.53	13.81
FUN_027569-T1			REP	MCM4, CDC54	0.25	0.03	9.24	12.49	10.68	14.18
FUN_006437-T1			cross	DNA pol delta	0.25	0.00	18.35	26.37	20.75	30.10
FUN_005656-T1			cross	FEN1, RAD2	0.25	0.01	25.10	36.81	28.38	42.17
FUN_006120-T1	HGT		BER	mutY/glycosylase	0.24	0.04	5.89	9.00	6.09	11.05
FUN_023002-T1^a^			REP	RNASEH2 A	0.24	0.05	11.04	17.00	12.63	19.14
FUN_017825-T1			NHEJ	POLL	0.23	0.02	17.88	17.72	20.05	20.22
FUN_015888-T1^a^		X	BER	PARP2_3_4	0.23	0.05	2.61	3.96	2.59	4.83
FUN_006214-T1			BER	high mob	0.22	0.02	725.00	924.10	814.96	1.031.40
FUN_002118-T1				apoptotic chromatin	0.22	0.00	26.56	29.50	29.85	32.68
FUN_008114-T1			REP	POLA1	0.22	0.00	15.22	17.46	17.02	19.15
FUN_023076-T1			related	von Will	0.21	0.01	21.18	20.21	26.47	19.72
FUN_012664-T1			NER	CUL4	0.21	0.00	15.36	18.94	17.39	20.20
FUN_010748-T1			cross	LIG1	0.21	0.01	11.62	16.26	12.52	17.95
FUN_025578-T1			NER	ERCC4, XPF	0.20	0.01	15.08	16.58	16.88	18.02
FUN_027905-T1			Alt. Ex. repair	SMC6	0.20	0.01	13.87	16.90	14.83	19.00
FUN_022565-T1			NER	ERCC5	0.20	0.00	16.56	21.05	18.25	22.66
FUN_020684-T1			BER	high mob	0.19	0.01	27.34	32.19	31.27	33.23
FUN_021992-T1		X	HR	RECQL5	0.19	0.00	11.48	11.21	12.69	11.70
FUN_012998-T1^a^			REP	POLG1	0.18	0.04	6.10	4.74	6.97	4.76
FUN_021263-T1^a^			NER	XPA	0.18	0.04	27.49	29.57	31.72	30.29
FUN_012179-T1		X	HR	RAD50	0.18	0.00	11.31	11.64	12.66	12.09
FUN_002457-T1^a^			NHEJ	Artemis	0.18	0.03	17.47	23.02	18.39	24.88
FUN_006977-T1			NER	RPB7	0.17	0.02	63.16	67.34	69.64	72.11
FUN_019131-T1			MR	MSH6	0.17	0.01	24.09	29.83	25.39	32.13
FUN_020752-T1^a^			cross	RFC1	0.17	0.02	14.68	13.95	16.35	14.04
FUN_008481-T1			BER	HMGB1	0.16	0.02	89.20	93.85	97.24	97.91
FUN_011948-T1^a^			NER	RPB2	0.16	0.02	23.79	23.53	25.02	25.00
FUN_016687-T1^a^		X	HR	MRE11	0.14	0.02	13.44	13.95	14.77	14.10
FUN_025669-T1^a^			HR	slx4	0.13	0.03	32.73	31.38	34.48	31.92
FUN_021347-T1^a^			NER	RPB3	0.13	0.04	17.43	19.51	18.15	20.16
FUN_021385-T1^a^			MR	MSH2	0.11	0.04	452.53	481.78	473.80	487.35

^a^Genes not validated by EdgeR

Notably, twenty-two genes characterized by a HSP Pfam domain (Hsp70, Hsp20/alpha crystallin family, Hsp90) and likely coding for Heat shock proteins are over-expressed in the Flight condition (*Additional file 3: Table S2*). While most of HSP genes over-expressed post X-ray radiation are characterized by the Hsp70 pfam domain, 14 here indeed show that domain, but 6 are characterized by Hsp20/alpha crystallin family, and 2 by the Hsp90 domain.

Moreover, 40 genes associated with antioxidant functions emerged as over-expressed (L2fc > 0 in Table 4*, Additional file 1: Fig. S9*) in the ISS environment. These genes spanned families such as thioredoxin, aldo/keto reductase, and glutathione S-transferase, among others. Within those with higher log2foldchange (L2fc > 0.33), we found three genes coding for SOD (1 SODC and 2 SODFe), two aldehyde dehydrogenases, three glutathione S-transferases, one thioredoxin, and one aldo/keto reductase. Conversely, 16 antioxidant genes were identified that displayed reduced expression in the ISS environment. Given the presence of genes previously identified as Horizontal Gene Transfers (HGTs) [30, 40] among the differentially expressed (DE) genes during spaceflight (Fig. 2A, light blue; Figs. S4, S10), we investigated whether these HGTs were enriched in the global transcriptomic response of the bdelloid *A. vaga* to the space environment. HGTs were significantly enriched in under-expressed DE genes with L2fc threshold of < 0 (13.28%; *χ*^2^ = 75.79, df = 1, alpha = 0.01). However, HGTs were not enriched in the overall pool of over-expressed genes set with a threshold > 0 (3.52%). Nevertheless, with threshold set at plus or minus 0.5 L2fc, HGT content was found to be enriched in both conditions: over-expressed genes (15.85%; *χ*^2^ = 6.15, df = 1, alpha = 0.01) and under-expressed genes (16.11%; *χ*^2^ = 47.73, df = 1, alpha = 0.01).

**Table 4:** Forty Genes predicted to code for antioxidants being over-expressed (Log2foldchange: L2fc > 0, FDR < 0.05) in Flight condition compared to Ground condition. The columns detail the gene ID, if they are identified as HGT, over-expressed the pathway in which the protein is involved, the log2foldchange, FDR, and transcripts per millions (TPMs) in Ground (G) and Fligh (F)t conditions for the two pools : Team 1 (T1) and Team 2 (T2). None of these genes were over-expressed in the core radiation response

Gene ID	HGT	Pfam ID	Pfam	l2cg	FDR	G_T2	G_T1	F_T2	F_T1
FUN_028564-T1		PF00248	Aldo/keto reductase family	0.54	0.00	3.84	2.94	6.06	4.36
FUN_007680-T1		PF00080	SOD Copper/zinc	0.47	0.00	334.00	457.85	424.80	644.32
FUN_020522-T1		PF00085	Thioredoxin	0.36	0.00	75.84	98.80	91.42	125.13
FUN_003320-T1		PF00081	SOD Iron/manganese	0.35	0.00	249.44	403.75	287.77	531.81
FUN_010925-T1		PF02798	Glutathione S-transferase	0.34	0.00	95.94	131.36	110.92	169.09
FUN_026222-T1		PF00171	Aldehyde dehydrogenase family	0.33	0.01	8.64	11.00	12.14	11.84
FUN_017932-T1		PF00081	SOD Iron/manganese	0.33	0.01	211.03	405.90	240.68	536.17
FUN_018831-T1		PF13417	Glutathione S-transferase	0.32	0.00	46.76	56.91	54.61	70.74
FUN_015944-T1		PF13410	Glutathione S-transferase	0.31	0.00	42.94	52.66	46.77	68.83
FUN_000688-T1		PF00171	Aldehyde dehydrogenase family	0.31	0.00	14.57	16.12	16.69	19.67
FUN_012693-T1		PF00578	AhpC/TSA family	0.31	0.00	103.23	116.62	118.78	144.02
FUN_005979-T1		PF00085	Thioredoxin	0.31	0.00	407.80	489.04	468.53	598.10
FUN_026682-T1		PF00248	Aldo/keto reductase family	0.29	0.00	21.52	33.00	25.35	38.45
FUN_005877-T1	HGT	PF00248	Aldo/keto reductase family	0.28	0.00	52.12	58.30	56.84	72.23
FUN_010924-T1		PF02798	Glutathione S-transferase	0.27	0.00	101.48	140.07	104.94	181.38
FUN_015891-T1		PF13410	Glutathione S-transferase	0.27	0.01	65.33	106.49	65.78	141.33
FUN_016659-T1		PF00248	Aldo/keto reductase family	0.26	0.00	22.71	30.79	25.45	36.07
FUN_027740-T1		PF00085	Thioredoxin	0.25	0.00	126.79	156.61	139.42	184.51
FUN_008045-T1		PF00085	Thioredoxin	0.24	0.01	53.32	68.26	57.34	82.31
FUN_017851-T1		PF02852	Pyridine nucleotide-disulfide oxidoreductase	0.24	0.01	33.78	38.50	37.05	44.84
FUN_012929-T1		PF00085	Thioredoxin	0.24	0.01	91.68	130.37	98.38	155.37
FUN_011027-T1		PF00248	Aldo/keto reductase family	0.23	0.04	9.97	9.47	11.38	10.76
FUN_000747-T1		PF00171	Aldehyde dehydrogenase family	0.23	0.01	9.23	11.33	10.06	13.03
FUN_008630-T1		PF00085	Thioredoxin	0.21	0.00	12.87	14.46	14.36	15.97
FUN_022296-T1		PF02852	Pyridine nucleotide-disulfide oxidoreductase	0.20	0.00	72.35	80.34	77.99	90.08
FUN_001986-T1		PF00085	Thioredoxin	0.20	0.05	30.22	37.37	34.09	40.13
FUN_005470-T1		PF00085	Thioredoxin	0.20	0.01	85.29	105.32	91.05	118.76
FUN_016398-T1		PF00248	Aldo/keto reductase family	0.18	0.01	52.36	61.72	56.63	67.30
FUN_018587-T1		PF00085	Thioredoxin	0.18	0.03	49.20	50.95	53.91	54.17
FUN_020404-T1		PF00248	Aldo/keto reductase family	0.17	0.01	139.88	170.27	155.91	178.45
FUN_006295-T1		PF00621	RhoGEF domain	0.17	0.02	58.24	67.51	59.89	76.34
FUN_008820-T1		PF00248	Aldo/keto reductase family	0.17	0.04	33.36	38.91	34.41	43.64
FUN_016496-T1		PF13409	Glutathione S-transferase	0.17	0.02	19.36	23.80	20.66	25.86
FUN_009077-T1		PF00578	AhpC/TSA family	0.17	0.00	162.81	165.42	174.92	177.45
FUN_021344-T1		PF00180	Isocitrate/isopropylmalate dehydrogenase	0.17	0.00	197.58	255.20	212.16	270.91
FUN_020429-T1		PF00085	Thioredoxin	0.16	0.00	43.71	50.38	47.70	52.68
FUN_026870-T1		PF00432	Prenyltransferase and squalene oxidase repeat	0.16	0.00	36.38	43.10	39.32	45.31
FUN_016583-T1		PF00085	Thioredoxin	0.15	0.03	19.30	22.90	21.22	23.55
FUN_021392-T1^a^		PF00171	Aldehyde dehydrogenase family	0.12	0.04	38.35	40.61	40.11	41.82
FUN_022706-T1		PF00085	Thioredoxin	0.12	0.00	138.17	152.20	144.44	155.53

^a^Gene not validated by EdgeR

### Differential gene expression in spaceflight: a comparison with responses to radiation

To broaden the transcriptomic analysis, the gene expression patterns in the ISS environment were compared with the general response to ionizing radiation (from [30]) that may be representative of key stress related genes in *A. vaga*. This core radiation response genes encompasses 906 over-expressed genes irrespective of radiation type (X-rays or Fe ions) or hydration status (Fig. 4), as outlined in [30]. Within this analysis, 68 genes were over-expressed in both the ISS and post-radiation contexts. Among these genes, 7 were associated with DNA repair (Artemis, APLF, PCNA, Rad50, PARP, Mre11, ATP-dependent DNA helicase, Table 4), and 6 coding for histones (Tables 4 and 5).

**Table 5:** Genes over-expressed both in the Flight condition and in the Core radiation response. The columns detail the gene ID, if they are identified as HGT, the predicted proteins (Pred.) involved in DNA repair, the predicted function based on blast search, the species and e-value of the search, the log2foldchange in the core radiation and in the Flight response

Gene ID	HGT	Pred.	Predicted function based on best blast hit	*Species*	e-value	Core	Flight
FUN_027504-T1						1.64	0.71
FUN_028525-T1^a^	HGT		Hypothetical protein DICPUDRAFT_33321	*Dictyostelium purpureum*	5.8e − 33	9.40	0.71
FUN_024631-T1^a^						5.01	0.54
FUN_015051-T1^a^						7.75	0.53
FUN_028031-T1						1.68	0.50
FUN_001706-T1^a^						3.01	0.49
FUN_019040-T1						1.80	0.48
FUN_009590-T1^a^						6.96	0.47
FUN_020814-T1			Disks large 1 tumor suppressor protein isoform X1	*Drosophila takahashii*	4.4e − 04	1.23	0.47
FUN_002284-T1			Toll-interacting protein-like	*Nicrophorus vespilloides*	3.1e − 24	0.84	0.47
FUN_001392-T1						2.32	0.46
FUN_019039-T1			Histone H3	*Adineta vaga*	4.1e − 65	1.99	0.42
FUN_001615-T1^a^	HGT		Hypothetical protein AK812_SmicGene10016	*Symbiodinium microadriaticum*	5.4e − 31	6.80	0.41
FUN_015235-T1^a^	HGT		Hypothetical protein AK812_SmicGene15751	*Symbiodinium microadriaticum*	2.4e − 45	5.70	0.39
FUN_001860-T1			Hypothetical protein	*Filimonas sp. YR581*	1.4e − 44	1.78	0.38
FUN_023539-T1^a^						3.96	0.38
FUN_018302-T1			Cytoplasmic polyadenylation element-binding protein 1 isoform X1	*Lingula anatina*	5.2e − 57	2.22	0.37
FUN_028871-T1						1.92	0.34
FUN_027187-T1			Diacylglycerol acyltransferase	*Brachionus koreanus*	6.3e − 109	1.07	0.33
FUN_007237-T1			hypothetical protein LOTGIDRAFT_106220	*Lottia gigantea*	3.8e − 27	2.15	0.33
FUN_016585-T1			NSFL1 cofactor p47	*Daphnia magna*	5.9e − 36	1.24	0.33
FUN_006663-T1		Artemis	Artemis	*Brachionus plicatilis*	3.9e − 48	5.88	0.33
FUN_023192-T1						2.47	0.33
FUN_002360-T1			Histone H3	*Adineta vaga*	4.1e − 65	2.11	0.32
FUN_002358-T1			Histone H2 Av	*Adineta vaga*	1.0e − 72	1.46	0.31
FUN_026163-T1		APLF	hypothetical protein DAPPUDRAFT_96117	*Daphnia pulex*	1.4e − 08	3.54	0.30
FUN_018160-T1	HGT		Hypothetical protein CCHL11_08714	*Colletotrichum chlorophyti*	8.2e − 154	0.83	0.30
FUN_006999-T1			Ribonucleoside-diphosphate reductase subunit M2 isoform X2	*Colobus angolensis palliatus*	3.6e − 140	2.88	0.28
FUN_006255-T1^a^			Smoothelin	*Folsomia candida*	1.2e − 29	1.02	0.28
FUN_016727-T1			LOW QUALITY PROTEIN: vacuolar protein sorting-associated protein 4B	*Geospiza fortis*	1.6e − 110	2.73	0.28
FUN_021724-T1			TNF receptor-associated factor 2 isoform X2	*Brachionus plicatilis*	1.5e − 18	3.46	0.28
FUN_002225-T1	HGT		Hypothetical protein PHAVU_005G0989001 g. partial	*Phaseolus vulgaris*	2.0e − 11	1.50	0.28
FUN_022535-T1			Predicted protein	*Hordeum vulgare subsp. vulgare*	0.0e + 00	1.14	0.28
FUN_017528-T1			Histone H2 A variant H2 Abd1 copy 2c. partial	*Adineta vaga*	3.5e − 76	1.91	0.27
FUN_027556-T1		PCNA	Proliferating cell nuclear antigen	*Brachionus plicatilis*	4.5e − 97	3.41	0.27
FUN_012031-T1			Unnamed protein product	*Vitrella brassicaformis CCMP3155*	2.4e − 68	1.98	0.26
FUN_025232-T1			U4 U6 small nuclear ribonucleo Prp31	*Brachionus plicatilis*	6.1e − 143	1.07	0.26
FUN_003621-T1			Histone H2 A variant H2 Abd1 copy 1a. partial	*Adineta vaga*	1.2e − 73	1.92	0.26
FUN_012464-T1			Hypothetical protein SPPG_03015	*Spizellomyces punctatus DAOM BR117*	6.5e − 52	1.05	0.25
FUN_012504-T1						1.28	0.25
FUN_003867-T1			SDR family NAD(P)-dependent oxidoreductase	*anaerobic bacterium MO-CFX2*	7.4e − 87	1.09	0.25
FUN_016499-T1			Programmed cell death 6-interacting protein-like isoform X1	*Pomacea canaliculata*	1.7e − 158	1.39	0.24
FUN_016234-T1			Transcription factor IIIB 90 kDa subunit-like isoform X1	*Brachionus plicatilis*	2.0e − 95	1.79	0.23
FUN_027622-T1^a^			Hypothetical protein BpHYR1_052278	*Brachionus plicatilis*	2.3e − 04	0.80	0.23
FUN_015888-T1^a^		PARP	Poly [ADP-ribose] polymerase 1-like	*Lingula anatina*	6.8e − 266	3.14	0.23
FUN_002222-T1			Dihydrofolate reductase	*Invertebrate iridescent virus 22*	4.9e − 24	0.82	0.22
FUN_012494-T1			IST1 homolog	*Mizuhopecten yessoensis*	1.2e − 33	1.65	0.21
FUN_003305-T1			E3 ubiquitin-protein ligase CHFR-like	*Orbicella faveolata*	7.5e − 72	2.77	0.21
FUN_020897-T1^a^			Iron-sulfur cluster assembly 1 mitochondrial	*Brachionus plicatilis*	2.3e − 69	1.39	0.20
FUN_020896-T1^a^			ER membrane protein complex subunit 8-like	*Branchiostoma belcheri*	1.8e − 37	1.11	0.20
FUN_003113-T1			Protein pelota-like isoform X2	*Centruroides sculpturatus*	6.7e − 130	1.19	0.19
FUN_012198-T1						2.49	0.19
FUN_021992-T1		DNA	atp-dependent dna helicase q5	*Brachionus plicatilis*	7.1e − 132	1.49	0.19
FUN_012179-T1		Rad50	Predicted protein	*Hordeum vulgare subsp. vulgare*	0.0e + 00	1.05	0.18
FUN_021821-T1^a^	HGT					1.91	0.18
FUN_025921-T1			Hypothetical protein C0Q70_06716	*Pomacea canaliculata*	2.7e − 131	1.10	0.18
FUN_007975-T1^a^			Histone H2 A variant H2 Abd2 copy A, partial	*Adineta vaga*	2.3e − 75	1.98	0.18
FUN_021882-T1			Hypothetical protein HELRODRAFT_184906	*Helobdella robusta*	9.2e − 25	1.82	0.17
FUN_003306-T1^a^			Lysine-specific demethylase 4B isoform X4	*Brachionus plicatilis*	1.9e − 115	2.02	0.17
FUN_022267-T1^a^			Coiled-coil and C2 domain-containing protein 1-like	*Aplysia californica*	8.0e − 64	1.14	0.17
FUN_016500-T1			Gamma-tubulin complex component 3 homolog	*Lingula anatina*	2.5e − 143	0.93	0.17
FUN_026293-T1			Hypothetical protein HELRODRAFT_73469	*Helobdella robusta*	5.2e − 20	2.63	0.16
FUN_022395-T1^a^			Sodium-dependent phosphate transporter 1	*Brachionus plicatilis*	8.8e − 141	3.21	0.15
FUN_020859-T1^a^			snf7	*Adineta vaga*	2.9e − 73	1.11	0.15
FUN_020918-T1^a^			Hypothetical protein CAPTEDRAFT_99754	*Capitella teleta*	5.2e − 78	1.75	0.15
FUN_016687-T1^a^		Mre11	Putative phosphorylase b kinase regulatory subunit alpha isoform X3	*Brachionus plicatilis*	0.0e + 00	1.44	0.14
FUN_007408-T1^a^			Hypothetical protein LOTGIDRAFT_230041	*Lottia gigantea*	1.4e − 06	0.83	0.14
FUN_007255-T1^a^			Transforming protein RhoA-like	*Saccoglossus kowalevskii*	1.5e − 94	1.01	0.13

^a^Genes not validated by EdgeR

On the other hand, 62 genes that were over-expressed in response to high level of radiation were under-expressed in the ISS environment (Fig. 4*;* Table 6). Some of these genes might be important to deal with radiation stress such as genes coding for an oxidoreductase, a dioxygenase, a transglycosylase, a von willebrand factor, a thioester reductase domain-containing protein, a PARP, protein argonaute-2-like (involved in RNA silencing processes), a 5′–3′ exoribonuclease, as well as many genes with unknown functions.

**Table 6:** Genes over-expressed in the Core radiation response but under-expressed in the Flight condition. The columns detail the gene ID, if they are identified as HGT, the predicted function based on blast search, the species and e-value of the search, the log2foldchange in the core radiation and in the Flight response

Gene ID	HGT	Predicted function based on best blast hit	*Species*	e-value	Core	Flight
FUN_015496-T1		Histidine–tRNA ligase, cytoplasmic-like isoform X2	*Nanorana parkeri*	9.0e − 77	1.24	− 0.93
FUN_023326-T1		SDR family oxidoreductase	*Archangium violaceum*	2.4e − 93	2.12	− 0.86
FUN_022325-T1		UMP-CMP kinase 2, mitochondrial	*Orchesella cincta*	1.5e − 22	0.88	− 0.70
FUN_015371-T1^a^	HGT	Hypothetical protein C0Q70_00159	*Pomacea canaliculata*	3.9e − 86	1.96	− 0.68
FUN_001780-T1					0.98	− 0.65
FUN_009164-T1					2.08	− 0.65
FUN_013932-T1					1.41	− 0.62
FUN_010960-T1	HGT	Hypothetical protein DICPUDRAFT_154763	*Dictyostelium purpureum*	9.4e − 28	1.73	− 0.61
FUN_023811-T1		Hypothetical protein MVEG_05746	*Mortierella verticillata NRRL 6337*	1.4e − 42	3.16	− 0.58
FUN_015290-T1		Sushi domain-containing 2-like	*Brachionus plicatilis*	2.5e − 88	2.35	− 0.53
FUN_028323-T1		Histone acetyltransferase p300 isoform X1	*Drosophila miranda*	6.5e − 158	0.85	− 0.53
FUN_012878-T1		Hypothetical protein RvY_15500	*Ramazzottius varieornatus*	2.6e − 40	2.38	− 0.52
FUN_023907-T1^a^		Uncharacterized protein LOC106181058 isoform X1	*Lingula anatina*	1.6e − 08	2.86	− 0.52
FUN_004340-T1^a^		Hypothetical protein Y032_0074 g817	*Ancylostoma ceylanicum*	1.8e − 58	2.67	− 0.51
FUN_017610-T1					1.69	− 0.50
FUN_012570-T1		Amino acid adenylation domain-containing protein	*Bacillus altitudinis*	3.0e − 36	4.13	− 0.50
FUN_028508-T1	HGT	Lytic transglycosylase domain-containing protein	*Hyalangium minutum*	1.4e − 25	2.19	− 0.45
FUN_013806-T1		Uncharacterized protein LOC105350353	*Fragaria vesca subsp. vesca*	3.4e − 23	2.35	− 0.45
FUN_004683-T1		Receptor-transporting protein 3-like	*Mizuhopecten yessoensis*	4.0e − 12	3.32	− 0.45
FUN_001797-T1	HGT	Hypothetical protein Glove_364 g8	*Diversispora versiformis*	3.7e − 43	1.93	− 0.45
FUN_004700-T1		Leucine-rich repeat containing proteins-like protein	*Adineta vaga*	5.2e − 29	1.76	− 0.44
FUN_009288-T1					1.61	− 0.43
FUN_017086-T1					2.08	− 0.43
FUN_003376-T1		Ras-like GTP-binding protein YPT1	*Aphanomyces invadans*	1.8e − 46	2.12	− 0.40
FUN_001708-T1		ypt homolog4	*Zea mays*	8.9e − 40	1.46	− 0.40
FUN_013952-T1		Dual specificity protein kinase TTK isoform X1	*Coturnix japonica*	2.2e − 20	1.37	− 0.39
FUN_010591-T1		3′−5′ exoribonuclease 1-like	*Ciona intestinalis*	2.2e − 48	1.04	− 0.39
FUN_028516-T1		Signal recognition particle 54 kDa protein-like	*Rhincodon typus*	7.7e − 199	1.96	− 0.38
FUN_023234-T1					1.86	− 0.38
FUN_019793-T1		Hypothetical protein MVEG_11384	*Mortierella verticillata NRRL 6337*	8.3e − 56	1.27	− 0.38
FUN_013960-T1^a^		NAD-dependent deacetylase sirtuin-2	*Penaeus vannamei*	1.7e − 87	1.71	− 0.37
FUN_006836-T1	HGT	Von willebrand factor type A (VWA) domain was originally protein	*Tetrahymena thermophila SB210*	2.4e − 34	3.53	− 0.36
FUN_001589-T1		Hypothetical protein AK812_SmicGene10016	*Symbiodinium microadriaticum*	4.5e − 48	1.91	− 0.35
FUN_024256-T1		Dynein heavy chain	*Achlya hypogyna*	7.2e − 12	2.39	− 0.35
FUN_019322-T1		Unnamed protein product	*Oikopleura dioica*	1.2e − 26	1.72	− 0.34
FUN_017042-T1		Hypothetical protein	*Absidia glauca*	2.8e − 11	1.13	− 0.33
FUN_013834-T1					3.30	− 0.32
FUN_023235-T1	HGT	Hypothetical protein SAMD00019534_055340	*Acytostelium subglobosum LB1*	6.0e − 248	2.64	− 0.32
FUN_017558-T1		Protein argonaute-2-like	*Amphimedon queenslandica*	3.2e − 53	1.28	− 0.31
FUN_017137-T1		Predicted protein	*Naegleria gruberi*	1.6e − 73	3.01	− 0.30
FUN_012747-T1		Hypothetical protein PHMEG_0008420	*Phytophthora megakarya*	5.2e − 13	1.78	− 0.30
FUN_024451-T1	HGT	Dioxygenase	*Brachionus plicatilis*	8.9e − 72	1.21	− 0.30
FUN_012571-T1		Thioester reductase domain-containing protein	*Thecamonas trahens ATCC 50062*	7.9e − 109	4.08	− 0.30
FUN_011005-T1		Hypothetical protein SAMD00019534_100260	*Acytostelium subglobosum LB1*	5.7e − 222	2.36	− 0.29
FUN_001360-T1		TPR repeat containing protein-like protein	*Philodina roseola*	1.1e − 44	1.66	− 0.29
FUN_012254-T1					1.33	− 0.29
FUN_001603-T1		Hypothetical protein	*Clostridium sp. AF18-27*	1.3e − 04	0.99	− 0.29
FUN_025989-T1		Uncharacterized protein LOC113049377 isoform X1	*Carassius auratus*	4.2e − 32	1.83	− 0.28
FUN_003863-T1		ras-domain-containing protein	*Fragilariopsis cylindrus CCMP1102*	1.5e − 87	1.46	− 0.28
FUN_023026-T1		Poly ADP-ribose polymerase 14-like protein	*Adineta vaga*	2.3e − 36	1.32	− 0.27
FUN_023572-T1		Uncharacterized protein LOC101235494	*Hydra vulgaris*	8.6e − 67	1.11	− 0.27
FUN_001181-T1		ABC transporter B family member 25 isoform X1	*Lingula anatina*	3.4e − 145	0.97	− 0.26
FUN_022710-T1		Nicotinate phosphoribosyltransferase	*Chlorobium sp. 445*	3.5e − 135	1.79	− 0.25
FUN_012769-T1	HGT	Hypothetical protein CAOG_00454	*Capsaspora owczarzaki ATCC 30864*	1.7e − 79	2.97	− 0.25
FUN_009111-T1					1.16	− 0.25
FUN_028143-T1	HGT	Hypothetical protein KFL_000880150	*Klebsormidium nitens*	7.2e − 40	1.72	− 0.24
FUN_004316-T1		Agrin-like isoform X2	*Sipha flava*	6.6e − 79	1.37	− 0.24
FUN_001688-T1	HGT	ADPribosylglycohydrolase superfamily protein	*Acanthamoeba castellanii str. Neff*	4.9e − 54	1.91	− 0.24
FUN_007618-T1		Fibropellin, partial	*Brachionus plicatilis*	6.9e − 15	1.36	− 0.21
FUN_022939-T1		Uncharacterized protein LOC106178153	*Lingula anatina*	1.1e − 98	1.80	− 0.21
FUN_018785-T1		Hypothetical protein CGI_10013828	*Crassostrea gigas*	2.9e − 149	1.42	− 0.16
FUN_003342-T1		Ubiquitin-conjugating enzyme E2 T-like	*Trichogramma pretiosum*	1.8e − 22	0.89	− 0.14

^a^Genes not validated by EdgeR

One of the enriched biological processes with the most abundant over-expressed genes is RNA metabolic processes (GO: 0016070) with 185 genes over-expressed in the Flight condition. Among these, only 4 genes were found in the core radiation response. However, we noticed that 56 of these genes were reported to be similarly over-expressed 2.5 h post X-ray radiation and post rehydration ([30], when using the same thresholds as Rob1: FDR < 0.05 and log2foldchange > 0 in DESeq2) (*Additional file 3: Table S2*). Among genes only found over-expressed in the Flight condition 15 genes are annotated with the Pfam domain Homeodomain (PF00046) and 10 genes with the domain “RNA recognition motif (PF00076), 16 genes with a tRNA synthesase pfam domain, various transcription factors, T-boxes, TATA box.

## Discussion

In the present study, a large-scale transcriptome analysis (RNAseq) was employed to assess the impact of microgravity in LEO on the biological processes in the bdelloid rotifer species *A. vaga*. Our RNAseq approach provided a hypothesis-free survey of all cellular pathways potentially influenced by the space environment.

### New passive hardware allowing autonomous culturing of hydrated bdelloids compatible with the KUBIK incubator on board ISS

A major innovation in this research is the development of new passive hardware, designed for autonomous culturing of hydrated bdelloid rotifers, which significantly reduces the need for astronaut intervention—a critical factor given the limited availability and busy schedules of astronauts aboard the ISS. This hardware was optimized for compatibility with the Kubik incubation system (ESA) and was tailored for bdelloid cultures by providing an initial food supply and ensuring adequate gas exchange. It accommodates a wide variety of bdelloid species, including both crawling specimens like *A. vaga* and free-swimming species such as *Philodina roseola*. Additionally, the hardware design incorporates features for rapid freezing of hydrated samples at − 80 °C. This fast-freezing function is crucial for capturing the real-time transcriptomic responses of space-exposed specimens. This is achieved with limited astronaut intervention and without the use of chemical products such as RNAlater which can have an impact on the gene expression levels in some cases [41, 42]. According to these parameters, our hardware ensures the successful post-thaw recovery of numerous individual rotifers, allowing for subsequent analyses on living specimens thereby extending the range of scientific questions that can be addressed in future experiments. Interestingly, the present hardware may be easily adapted for other experiments. Indeed, the use of PL30-2G ensures high compatibility with other biological specimens such as human cell cultures. By modulating the storage temperatures, it was possible to reduce the metabolic rate of the rotifers prior to loading into the Kubik incubator. This was crucial for extending the viability and ensuring absence of stress in *A. vaga* samples at the time of freezing of the individuals during the flight experiment. Preliminary ground experiments validated an absence of stress (i.e., presence of tun/contracted individuals) in specimens stored at 10 °C for 2 weeks, subsequently exposed to a maximum of 15 °C for another 2 weeks. By contrast, a temperature of 19 °C, while commonly used for laboratory cultures, only sustained similar conditions for approximately 7 days (Data not shown). Therefore, the 15 °C culturing temperature was selected to ensure flexibility of the space experiment characterized by specific constraints, such as limited crew availability and the potential launch delays.

### Successful exposure of hydrated bdelloid rotifers onboard the ISS: modulation of stress-related and unknown-function genes

Bdelloid rotifers were successfully cultured at the end of 2019 on the ISS environment without any recorded technical issues or significant deviations from the planned experimental design. *A. vaga* individuals were autonomously cultured and exposed to 12 days of microgravity, of which 7 days of cultivation in the KUBIK facility at a constant temperature of 15 °C (Fig. 1D). Simultaneously, a ground-based experiment was executed, mirroring the temperature profiles and timelines of the space samples, except for the microgravity and higher radiation levels (see below). Importantly, both flight and ground experiments were performed in synchrony on the same batch of *A. vaga* culture, thereby strengthening the validity of the comparative analyses. A notable outcome from both flight and ground controls was the presence of eggs, indicative of effective bdelloid reproduction during the experiment. In the absence of visual confirmation from flight samples, this finding supports the notion that space-exposed samples remained reproductively active, undeterred by the microgravity conditions. Future research efforts will use onboard microscopy techniques to further examine whether microgravity impacts the activity levels of these rotifers, or if discernible differences exist in the stress levels (fraction of contracted/tun shape individuals for example) of individuals when comparing space-exposed specimens to ground controls. This will pave the way for a more comprehensive understanding of the biological effects of space travel on microscopic animals. For example, a previous experiment on *C. elegans* indicated that spaceflight significantly affected swimming frequency, slowing the movement of the worms under microgravity [43].

During the 9-day stay aboard the ISS in LEO, hydrated individuals of *A. vaga* were exposed to an absorbed dose of 3.94 mGy, with a total dose equivalent of 8.49 mSv. Radiation data from the launch to ISS berthing are not available. To provide a comparative frame of reference, the global average effective dose from natural background radiation at the surface of Earth is approximately 2.4 mSv per year (source United Nations Scientific Committee on the Effects of Atomic Radiation, UNSCEAR, 2000). If the observed radiation levels from the space mission were extrapolated over a year period, the calculated dose equivalent would rise to 344 mSv which is approximately 143 times higher than typical terrestrial exposure levels. Despite this elevated exposure, the observed dose values are markedly below the species’ extreme radiation tolerance levels, which range between approximately 5000 and 7500 Gy [26]. While microgravity is anticipated to be the primary factor influencing the transcriptomic response of the space-exposed samples, it is important to consider the potential contributory effects of galactic cosmic rays (GCRs) on the observed transcriptomic patterns in the experimental setup. This could be further validated in future experiments beyond low-Earth orbit, when spaceflight platforms become available, for example in lunar orbit on the planned Gateway which is scheduled to spend a significant proportion of its orbit outside of the Earth’s magnetosphere; therefore, an increased exposure to GCRs is anticipated.

RNA was extracted and sequenced from 16 biological samples—eight from flight conditions and eight from ground control conditions. Quality control metrics supported the high-quality nature of the sequencing data, rendering it suitable for subsequent transcriptomic analyses. Unexpectedly, PCA analysis revealed that the transcriptional response was influenced by the animal pools used for the experiment, a surprising finding given that bdelloid rotifers reproduce asexually. Each pool originated from randomly selected cultures of *A. vaga*, all stemming from a unique clone and were cultivated synchronously under similar conditions. These pools were prepared in parallel by two different teams, both trained identically and following the same protocol. The underlying cause of this variability remains elusive, underscoring the need for rigorous replication in such studies. Notably, microbial communities from the initial petri dishes may have shaped the transcriptional landscape of the bdelloids, potentially affecting feeding habits, immune responses, or mobility, despite no observed macroscopic differences in controls. To mitigate this variability, eight replicates were used for both flight and ground conditions. The applied bioinformatic pipeline used effectively accounted for the variability between pools and was specifically designed to isolate and distinguish the effects of the space environment on gene expression. The data revealed that 18.61% of the *A. vaga* transcriptome was differentially expressed when exposed to the conditions aboard the ISS. Specifically, 10.71% of genes were upregulated and 7.90% were downregulated. This data suggests that the organism’s genetic activity alters in response to space environment. Most differentially expressed genes presented a log2foldchange between 0.01 and 0.5. Additionally, 82 genes were observed with a log2foldchange greater than 0.5, and 596 genes had a log2foldchange less than − 0.5. At first view, transcriptomic data suggest a nuanced response to space environment while most changes were moderate.

Despite the availability of the *A. vaga* genome, a substantial portion remains either unannotated or annotated with non-specific functions [30, 40]. This presents a significant limitation, as genes of unknown function are inevitably excluded from this study, leaving potential insights untapped in the absence of further genomic characterization for *A. vaga*. Nevertheless, to gain deeper insights into the molecular functions impacted by spaceflight, we performed a GO enrichment analysis on differentially expressed (DE) genes identified in *A. vaga* during the space experiment compared to ground reference samples. Among genes differentially expressed during space flight, a significant enrichment in translation processes (GO:0006412) was reported and could be interpreted as an increased demand for protein synthesis. In parallel, protein folding (GO:0006457) also shows substantial enrichment, confirming the protein synthesis processes.

In line with these observations, we also detect an enrichment in processes related to DNA replication (GO:0006270 and GO:0006260). This may be associated with DNA repair mechanisms or linked with the reproductive activity of bdelloid specimens during spaceflight. Indeed, many DE genes (66) slightly over-expressed have functions related to DNA replication/repair. Another hypothesis may be the possible link with an increased activity of nurse nuclei that undergo multiple cycles of post developmental endoreplication during oogenesis [39]. The significant enrichment of DNA replication-related genes among over-expressed genes has not been previously observed under other stress conditions, such as exposure to high or low LET (Linear Energy Transfer) radiation [30]. This observation suggests that microgravity may uniquely affect cell cycle regulation in bdelloid rotifers. In *A. vaga*, DNA replication is typically confined to developing oocytes or nurse cells, given that bdelloid rotifers are eutelic. Although DNA replication can also be associated with DNA repair, we did not observe a similar enrichment following radiation-induced double-strand breaks [30]. The upregulation of DNA repair genes in the spaceflight samples indicates that microgravity may elicit a distinct molecular response compared to radiation exposure.

In addition to these processes, pathways related to RNA metabolism emerged prominently in our analysis. This is complemented by enriched terms such as mRNA splicing (GO:0000398) and RNA metabolic processes (GO:0016070), emphasizing the critical role of RNA processing and metabolism in cellular adaptation to spaceflight conditions. Noticeably, we observed a significant overrepresentation of genes associated with RNA processing, metabolism, and splicing pathways, with 185 genes identified in our study. If some of them are also found post X-ray radiation, indicating similar transcriptomic response, others seem specific to the stress due to space environment. This finding highlights the sensitivity of RNA-related processes to microgravity, suggesting a reconfiguration of the transcriptional machinery to meet the unique challenges of the spaceflight environment. Previous studies have similarly observed the impact of microgravity on RNA metabolic processes, including changes in splicing and RNA transport pathways in both plants and mammals, underscoring the relevance of these processes to spaceflight adaptation. However, this aspect remains largely underexplored and may be of interest for future investigation [44–46].

Furthermore, enrichment in protein catabolic processes (GO:0030163, GO:0006511) may imply a heightened turnover of damaged or unnecessary proteins, whereas gluconeogenesis (GO:0006094) could reflect shifts in energy metabolism. Endomembrane system functions, such as intracellular protein transport (GO:0006886) and ER-to-Golgi vesicle-mediated transport (GO:0006888), were also enriched, suggesting cellular reorganization or heightened secretory activity. Finally, pathways involved in cell redox homeostasis (GO:0045454) and signaling (GO:0023052) also appear enriched, suggesting an intricate interplay of intracellular signals to maintain cellular balance under the oxidative and radiation stress typical of space environments. The 40 genes coding for antioxidants being slightly over-expressed in the flight conditions (Table 4) indicates the potential induction of oxidative stress by spaceflight. Particularly, we found three SOD and one thioredoxin to have high expression levels in the flight condition (TPMs > 400). Antioxidant gene expression has been shown to be impacted in the tardigrades *Paramacrobiotus richtersi* and *Ramazzottius oberhaeuseri* exposed to space environment with an increase in glutathione enzymatic activities, and decrease of expression of catalase and superoxide dismutase [47].

On the other hand, down expressed genes during spaceflight were notably enriched in GO related to protein ADP-ribosylation (GO:0006471), suggesting a potentially decreased capacity for DNA repair and stress response. Indeed, ADP-ribosylation is a post-translational modification where ADP-ribose is attached to proteins. This process is mediated by enzymes like PARPs (Poly ADP-ribose polymerases). PARPs are activated in response to DNA damage and play a critical role in the DNA damage response pathway. The down expressed genes response was also associated with an enrichment in proteolysis (GO:0006508) that may affect the turnover of damaged or misfolded proteins, potentially impairing cellular maintenance.

To deepen the understanding of stress-related genes potentially influenced by spaceflight, the transcriptional response of *A. vaga* exposed to the ISS environment was compared to core radiation genes described by Moris et al. [30] (Fig. 4). Among the core set of 906 radiation-responsive genes, 68 were concurrently upregulated in both ISS-exposed and post-radiation contexts, including key players in DNA repair mechanisms such as Artemis, APLF, PCNA, Rad50, Mre11, and PARP. Interestingly, 62 genes typically upregulated in response to radiation were downregulated during spaceflight exposure. This observation may imply a risk to *A. vaga* genomic integrity or at least merits further investigation. Indeed, the impact of spaceflight on genomic integrity remains an open question. Given the exposure to CGR, maintaining DNA integrity emerges as a critical adaptive requirement for prolonged space travel. The upregulation of 68 genes specifically involved in DNA repair pathways such as HR, NHEJ, BER, and MR suggests that some form of genomic stress is indeed induced by spaceflight. Notably, only three genes involved in DNA repair were downregulated and these do not play a part in the core radiation response, implying that such repair mechanisms are largely active during space travel. However, caution is advised, as the radiation dose in the ISS environment is much lower as well as chronic of nature, which is different than the experimental conditions previously applied to both desiccated and hydrated bdelloids. Further research is essential to confirm the relevance of these repair mechanisms in the ISS environment.

Bdelloid rotifers have acquired a diverse array of genes from non-metazoan organisms through HGT, accounting for 8.3% of all genes in *A. vaga*. These genes seem crucial for their evolution, providing bdelloids with novel biochemical functions that enable them to adapt to environmental challenges [48, 49]. Identified functions include desiccation tolerance, nutrient exploitation, DNA damage repair, and more recently, defense against fungal pathogens [50]. In this study, a significant enrichment of HGT was observed in genes that exhibited pronounced differential expression in response to spaceflight (log fold change >|0.5|). These genes do not have evolutionary ties to adaptations for environments like microgravity. Nevertheless, our results indicate that HGT is instrumental in the adaptive responses of bdelloid rotifers to various environments, including those encountered on the ISS. The specific roles of these HGT-driven changes—whether increased or decreased in expression—remain unclear. However, our findings support the hypothesis that these genes such as Mannan endo-1,4-beta-mannosidase A and B, proteasome activator complex are deeply integrated into bdelloid metabolism and likely undergo modifications to aid their adaptation to environmental stresses.

### The stone age of bdelloid space research: future research perspectives

After 12 days in the microgravity and space radiation conditions of the ISS, the bdelloid rotifer *A. vaga* populations exhibited changes in the expression of 18.61% of their genes. Interpreting data from space experiments presents unique challenges, especially due to technical constraints that limit the number of independent replicates, which are common in terrestrial studies. Previous studies with established model organisms, such as *Arabidopsis thaliana* and various bacteria, have shown significant variations in outcomes between individual space experiments. These variations, influenced by different equipment and methodologies [12–14], underline the necessity of conducting multiple independent experiments.

The impact of spaceflight on transcriptome-level responses in small metazoans remains limited. Future data from missions like The Cell Science-04 (CS-04) mission, investigating the transcriptomic response of the tardigrade *Hypsibius exemplaris* during spaceflight in 2021, will provide valuable insights into potential common microgravity responses in small metazoan polyextremophiles. Currently, only *C. elegans* offers robust transcriptomic data from multiple spaceflights, enabling comparative studies to explore the effects of spaceflight on gene expression. In *C. elegans*, transcriptomic responses have highlighted conserved pathways, such as the unfolded protein response, with heat shock protein (HSP) genes like *hsp-16.1* and *hsp-70* consistently differentially expressed [51]. Similarly, we noticed some HSP-related gene upregulated in *A. vaga* during spaceflight potentially suggesting conserved mechanisms among small metazoans and warranting further exploration with varied microgravity exposure durations to isolate core transcriptomic responses.Bdelloid rotifers, being microscopic and easy to culture, can be replicated many times. In this study, a greater number of replicates were used to overcome the limitations of ISS experiments. Ensuring the reproducibility of these findings is vital for verifying their accuracy and exploring a range of parameters and flight scenarios. For instance, studying the effects of slightly higher cultivation temperatures on rotifers could reveal more significant transcriptomic changes, potentially due to increased metabolic activity. Our findings lay crucial groundwork for future research aimed at validating and extending these results. Understanding the long-term and generational impacts of space conditions on bdelloid rotifers is crucial for advancing space biology. While direct space experiments are limited, ground-based analogs, such as the Random Positioning Machine (RPM), may offer valuable insights. However, accurately replicating the combined effects of microgravity and space radiation is challenging [52–54]. In particular, research has shown conflicting results concerning the DNA damage response (DDR) in various organisms under spaceflight conditions, with some studies reporting no significant effects, while others have observed enhanced or suppressed DDR [54]. These inconsistencies, particularly regarding the impact of microgravity on DDR observed in ground versus space experiments, might be attributed to the limitations of ground-based microgravity analogs in fully replicating space conditions.

This study here represents the first in a series of ESA-supported experiments investigating the impact of microgravity on bdelloid rotifers. A follow-up experiment in 2020 aimed to compare the DNA repair kinetics of rehydrated bdelloids post-irradiation in normal gravity (1G), microgravity (µG), and simulated gravity (1G). After the flight, genomic data were extracted from eggs or juveniles produced during the spaceflight. By employing whole genomic sequencing, we aim to compare the genomic structure of these specimens with the pre-exposure genomic data of *A. vaga*. This comparative analysis will enhance our understanding of DNA repair in space, particularly regarding the potential decrease in DNA repair efficiency in microgravity. Conversely, if *A. vaga* individuals maintain efficient DNA repair during spaceflight, it will shed light on the molecular processes involved in DNA repair in these radiation-tolerant animals.

At short term, a new experiment is planned to analyze the morphology, activity, and behavior of rehydrated bdelloids aboard the ISS. Additionally, an astrobiology experiment is under development to expose desiccated bdelloids to the deep space vacuum and cosmic radiation. Together, these experiments are expected to significantly advance our understanding of the mechanisms involved in protecting from and repairing radiation-induced damage. These investigative efforts pave the way for the discovery of novel molecules or potential candidate genes [30, 37] that may be instrumental in enhancing health span. They hold promise for safeguarding astronauts and others exposed to radiation during spaceflights or medical treatments. Equally important, this research serves as a steppingstone in the field of exobiology, delving into the profound questions surrounding the origin of life and its capability to endure and proliferate in the harshness of outer space.

## Conclusions

This study presents the development and successful deployment of new passive hardware compatible with the ESA KUBIK facility onboard the ISS, enabling the autonomous cultivation of hydrated bdelloid rotifers *A. vaga* under spaceflight conditions. Over a 12-day period, these animals were exposed to the combined effects of microgravity and increased radiation levels characteristic of LEO, with parallel ground controls maintained under equivalent thermal conditions. Transcriptomic analyses revealed that 18.61% of *A. vaga* genes were differentially expressed in response to spaceflight. Although the magnitude of expression changes (log2foldchange) was generally moderate compared to previous studies involving acute ionizing radiation exposure, the expression of several key genes known to be involved in the bdelloid core radiation response significantly changed. Notably, genes associated with protein synthesis, RNA metabolic processes, and DNA repair pathways were upregulated in the spaceflight condition. Unexpectedly, our findings also reveal a significant enrichment of horizontally acquired genes (HGTs) among both over- and under-expressed transcripts, suggesting a potential role for HGTs in the adaptive response of bdelloid rotifers to atypical environments such as space. These results establish a baseline dataset for future experiments aimed at exploring bdelloid responses to space environments, including studies on genome evolution, behavioral adaptation, and survival in deep space conditions.

## Methods

### Experiment hardware for bdelloid ISS space exposure

Experiment-specific hardware was designed and built to expose hydrated *A. vaga* individuals to the ISS environment (Fig. 1A, B). The hardware ensured the adequate culturing conditions and storage of *A. vaga* individuals on board the ISS, utilizing a specialized system. The key components are:

- (1) The Culture Bag (CB), a PL30-2G bag, designed by OriGen Biomedical, used for housing the rotifers. Constructed from inert Fluoro-ethylpolymer (FEP), the PL30 bags enable gas diffusion. Filled with 17.5 mL of Spa water and 2.5 mL (12.5% v/v) of sterile lettuce juice as a food source (produced as in [30]), this bag has been validated for autonomous cultures throughout the entire mission phase, including integration, launch, docking, on-board storage, cultivation, and freezing at − 80 °C.
- (2) The Experiment Container (EC): Known as the KIC Magnum NLA, this container accommodates 5 CBs. Crafted from high-resistance aluminum alloy, it functions as the mechanical interface for the CBs as well as to the KUBIK facility (see (5)) on board ISS. Its design includes a protective anodized finish, and a pattern of holes covered by an adhesive patch to prevent classification as a pressure vessel. The pattern of holes allows gas exchange with the external environment. Two ECs were sent to the ISS. Two additional ECs were used for ground reference experiment.
- (3) The Internal Frame: This component is manufactured from aluminum alloy AA 6068 and serves to hold five CBs securely within each EC. The specific choice of material is aimed at aiding the quick freezing of samples. The internal frame consists of six segments, connected by spacers and screws.
- (4) The Experiment Hardware (EH): This collective term refers to the integrated CBs within the EC, forming a comprehensive system for the autonomous cultivation of *A. vaga* individuals.
- (5) The KUBIK Incubator: The hardware is designed to be inserted into the KUBIK incubator available on board the ISS, facilitating the exposure of the culture to 15 °C for a cultivation period of 1 week.
- (6) Temperatures of flight and ground controls were monitored by 4 ibuttons (MAXIM iButton DS1922L).

### Implementation of the rotifer in space experiment

A detailed illustration of the flight timeline can be found in Fig. 1D.

#### Establishing laboratory cultures of A. vaga

Experiments were performed using isogenic *A. vaga* clones derived from a single individual from the laboratory of Matthew Meselson at Harvard University [40]. A set of *A. vaga* individuals initially cultivated in UNamur URBE laboratory were transported to the Kennedy Space Center (KSC) facilities 1 month before launch. Cultures were maintained in large petri dishes at 21 °C, hydrated with natural spring water (Spa), and fed with sterilized lettuce juice. All sample preparatory activities were conducted under a laminar flow. Every 2–3 days, cultures were individually inspected under a binocular microscope for health assessment, fed with 500 µL of sterile lettuce juice, and plates with a high density of active, uncontaminated animals were selected to produce new cultures. After 2 weeks of cultivation, a total of 45 culture plates were selected to be used for flight and ground experiments. The day before the experiment flight integration, selected cultures received an additional 500 µL of sterile lettuce juice which ensured the creation of consistent and contaminant-free populations suitable for control and spaceflight experimentation.

#### Collection of A. vaga individuals for integration

On the day of integration (2nd December 2019), 30 out of 45 plates of *A. vaga* individuals were selected for inclusion in flight and ground hardware, with 15 plates randomly assigned to two integration teams. Each plate was rinsed with sterile water (Spa) to remove residues, eggs, and dead animals. The living organisms were then collected through the addition of 450 µL of 5 M NaCl and vortexed, followed by transferring the supernatant containing the rotifers to Falcon tubes. After centrifugation (3220 G, 10 min, 4 °C), the supernatant was discarded, and pellets from each tube were combined into a single tube. Tubes were then rinsed with water (Spa) to ensure maximum collection of the rotifers, followed by two additional rounds of centrifugation and rinsing with water (Spa) to remove all the remaining NaCl. The number of collected animals was evaluated for each team by counting the organisms in a 2 µL volume, taken from a final volume of 2 mL, with six independent counts conducted. All these procedures were executed under a laminar flow, except for the counting, which was performed near a Bunsen burner. Next, 10,000 *A. vaga* individuals were carefully loaded into the PL30-2G (OriGen Biomedical) using a micropipette. They were suspended in 20 mL of sterilized water (Spa) mixed with 12.5% lettuce juice, which had been filtered using a 2 µM filter. Air was meticulously removed from the bag to prevent air bubbles, which might interfere with the experiment as they could lead to the partial or complete desiccation of some of the animals. Before being placed in the ECs, each PL30-2G underwent a successful leak test. This test involved exposing the samples to vacuum to detect any potential leaks in the bag, ensuring the hardware’s safety during transportation to the ISS. After the leak test and before the final integration into the hardware, the morphology of the bdelloids inside the PL30-2G was inspected with binoculars to confirm that the animals had not been harmed or stressed in the preceding procedures (*Additional file 1: *Fig. 1A–D). In normal conditions, *A. vaga* individuals are crawling in leach-like movements, while in stress conditions they adopt typical tun/contracted shape.

#### Overview of the flight and ground experiment

In an effort to account for potential biological variability among our samples, the experimental design focused on two distinct pools of samples. These were cultivated and exposed to identical conditions but processed by two independent teams. Through this approach, we sought to include possible variability stemming from the manipulation of rotifers during the collection and integration process, and to account for the inherent biological variability between two *A. vaga* populations that were theoretically similar (Fig. 2A).

*A. vaga* individuals were gathered and placed into CBs on December 2nd 2019. Out of a total of 25 loaded PL30 bags, 10 were randomly chosen for flight exposure (five for each CB) and another 10 were selected for a ground reference experiment (GRE). The GRE samples were housed inside a climatic chamber that simulated the thermal history of the flight samples as accurately as possible. The remaining CBs were cultivated in a manner akin to the GRE-CB and served as a visual control to check the activity of bdelloid cultures throughout the experimental process.

Once the experiment integration was completed, the two flight Experiment Containers (ECs) were preserved at 10 °C and transported to the ISS on board a SpaceX Falcon-9 rocket (CSR-19 mission) on December 5 th, 2019, launching from NASA’s Kennedy Space Center in Merritt Island, FL, USA. Upon arrival on board the ISS on December 8 th, the samples were kept at a temperature between 9.5 and 12.5 °C within an onboard refrigerator (MERLIN), prior to being installed into the two KUBIK incubators (ESA) (10 th December 2019), which were preset to 15 °C. Inside the KUBIKs, the bdelloids were cultivated for 1 week at a steady temperature of 15 °C. All activities on board the ISS were performed by the astronaut Luca Parmitano. At the conclusion of the experiment (17 th December 2019), the samples were fixed by freezing, accomplished by placing the ECs inside the MELFI hardware. The corresponding GRE samples were simultaneously inserted into a conventional laboratory freezer at − 80 °C to mirror the conditions of the flight samples. For their return, the ECs were packed into a NASA-provided “Double Coldbag” and transported back to Earth (7 th January 2020) via SpaceX CRS-19 (the same vehicle used for upload), then transported to the UNamur (arrival on the 14 th January 2020) URBE laboratory in Belgium for disassembly (22nd January 2020), accompanied by the ground controls.

#### Collection of A. vaga individuals post flight

Following hardware disassembly, the PL30 samples (both flight and ground) were stored at − 80 °C to preserve the integrity of the biological material. For proper RNA extraction from eight *A. vaga* samples, a specific collection procedure was employed. Each PL30-2G was removed from the freezer, sealed in a Zip bag, and immersed in a 56 °C water bath for 2 min. Using a sterile syringe (designated one per collection), 20 mL of liquid was extracted from each thawed PL30, and then divided into two separate sterile Falcon tubes, kept on ice throughout the collection process. The pouch was subsequently rinsed with 10 mL of water (Spa), and the collected liquid was evenly distributed in the two tubes. The rotifers were then pelleted through centrifugation for 30 s at 805 G and 4 °C. The supernatant, containing culture debris and some rotifers (to be used later for living rotifer verification), was removed. The pellets were washed with 15 mL of ice-cold spa water and centrifuged for 7 min at 3220 G and 4 °C, followed by supernatant removal. The cleaned rotifer-containing pellets were then transferred to a sterile 1.5-mL DNAse- and RNAse-free microcentrifuge tube, with the collection tubes receiving a final rinse with 500 µL of spa water. The microcentrifuge tube was centrifuged at 16,000* g* for 1 min at room temperature, and after supernatant removal, the rotifers were fixed in liquid nitrogen, securing the biological material for subsequent analyses. The whole procedure, from the melting to the fixation in liquid nitrogen, took maximum 15 min per sample.

### Radiation level and temperature recording

DOSIS 3D Hardware: The scientific aim of the DOSIS 3D experiment was to monitor the radiation environment inside the Columbus Laboratory on board the ISS with active and passive radiation detectors at fixed locations. Data was gathered by the active radiation detectors for the time of the mission (7 th–16 th December 2019).

#### iButtons

Temperatures were monitored using iButtons (DS1922L) located on the RoB1 hardware and activated at the end of the integration procedure. A temperature range from minus 40 to plus 85 °C could be monitored according to technical specifications of iButtons. Temperature measurement accuracy was ± 0.5 °C from − 10 °C to + 65 °C. Temperature was measured each 15 min, starting from the complete hardware integration. Both flight and ground hardware were screened using dedicated iButtons. Temperature profiles of the two flights hardware (Flight#11 and Flight#12) and the two ground controls hardware (Ground#21 and Ground#22) are provided in *Additional file 3: Table S3*.

### RNA extraction, quantification, and sequencing

Total RNA was extracted using the RNAqueous-4PCR Kit (Ambion, Austin). Lysis was performed using 500 µL of lysis buffer (RNAqueous-4PCR Kit, Ambion, Austin) for a complete duration of 30 min. RNA samples were treated with DNAse (Ambion, Austin) and quantified using a Qubit Fluorometer (ThermoFischer) and Nanodrop (ThermoFischer). A minimum of 60 µL was collected from each sample and delivered to Genomicscore (Leuven, Belgium) for RNA sequencing. RNA libraries were prepared using the TruSeq Stranded mRNA protocol (Illumina, San Diego, USA). Libraries were purified and evaluated using an Agilent 2100 bioanalyzer. RNA libraries were sequenced with an Illumina NextSeq 500 (Illumina, San Diego, CA USA) as paired-end 2 × 75 base pair read using the NextSeq version 2.5 mid or high-output 150 cycle kit (Illumina). The fastq files of each transcriptome are accessible on NCBI under the Bioproject PRJNA1142504 under the biosamples SAMN42945155—SAMN42945170 [55].

### Bioinformatic and data analysis

#### Differential gene expression analysis (DGE)

The whole bioinformatic workflow described below was performed in Galaxy Europe platform (usegalaxy.eu) as described in Moris et al. [30]. Briefly, sequencing adapters and the first ten nucleotides were removed with Trim Galore (version 0.4.3.0) (http://www.bioinformatics.babraham.ac.uk/projects/trim_galore/). Before and after trimming the adapters, the quality of the transcriptomes was checked by FASTQC evaluation software (version 0.67) (http://www.bioinformatics.babraham.ac.uk/projects/fastqc/). Trimmed-reads were mapped onto the genome assembly of *A. vaga* [56] using RNA-Star (version 2.5.2b0) [57]. For the mapping, the gff file was transformed into a gtf file using gffread (version 2.2.1.1) [58]. A matrix of normalized counts per gene was assembled by htseq-count (version 0.6.1) [59].

The differential gene expression analysis (DGE) was conducted using DESeq2 (version 2.11.39 [60]) considering the presence of a batch effect. For subsequent analyses, genes were considered significantly differentially expressed if they had FDR < 0.05 for DESeq2 analyses. The DGE analysis was confirmed using EdgeR with batch effect (version 3.28.0; [61]) in the R statistical software (version 3.4.1) (http://www.R-project.org). MA and Volcanic plots were carried out in R (version 4.4.3) using the package EnhancedVolcano plot (version 1.20) [62]. The Base Mean (*z*) represented on differential plots (*y* axis) was transformed as follows: − log(1/(*z* + 1) and is therefore proportional to the base mean values on the plots. For example, a base mean of 1000 is represented by the value 3 on the plots, while a base mean of 10 is represented by the value 1 on the plots.

#### Transcripts per millions

Transcripts per million (TPMs) for each gene were computed with a custom Python script (available on https://github.com/vmoris/TPM_script) [63].

#### Annotations

Functions of genes (including DNA repair and antioxidant genes) from the *A. vaga* genomes were previously identified in Moris et al. [30]. More precisely, Gene Ontology (GO) ids, Pfam domains, and KEGG ids were annotated using InterProScan in Galaxy with *A. vaga* gene set [64]. We searched for putative protein functions by similarity searches using BLASTP in the non-redundant database (NRdb) using a threshold expect value E < 10 − 5.

Horizontal gene transfer (HGT) acquisition was previously identified in Moris et al. [30] based on Alienomics [40]. This approach only identifies putative HGTs from non-metazoans (e.g., bacteria, plants or fungi).

#### Gene Ontology enrichment analysis

GO enrichment analysis was performed in R, using the package “topGo” from Bioconductor [65].

#### HGT enrichment analysis

Chisquare tests were carried out to test whether the number of HGTs found to be over-expressed or under-expressed was significantly different from the expected percentage of HGTs in *A. vaga* genome (8.3% genes; [40]).

## Supplementary Information

Additional file 1: Figures S1-S10. Fig. S1 Overview of random A. vaga individuals loaded in PL30beforeand after leak testduring integration. Animals remain active after leak test. No modification of behavior was reported. Captured using Zeiss Stemi 305 Binoccular coupled with Canon camera. Fig. S2 Picture showcasing A. vaga individuals stored under conditions mirroring ground controls, including hardware. Captured immediately prior to sample fixation on December 17, 2019, the picture confirms:no detectable contaminants,typical activity in hydrated bdelloids, andegg presence within autonomous cultures. Captured using Zeiss Stemi 305 Binocular coupled with Canon camera. Fig. S3 Venn diagram representing the results obtained with DESeq2 and EdgeR. The genes being over-expressed with Deseq2, with EdgeR, and under-expressed with DESeq2and with EdgeR. Fig. S4 Volcano plot of genes with lowest and highest log2foldchange comparing flight and ground condition. Genes under-expressed in flight conditionare colored in dark blue while those over-expressedare colored in green. Genes differentially expressed and involved in DNA repair are colored in magenta, those coding for antioxidants in orange and identified as HGTs in light blue. Genes non-significantly differentiallyexpressed are colored in gray. Fig. S5 Frequency plots representing the number of genes with GO ids for a specific log2foldchange values identified as A) over-expressed genes or B) under-expressed genes, with a specific l2fcbeing characterizedor notwith a Gene Ontology term. Fig. S6 Differential plot showing the 25 top ranked genes with highest log2foldchange values among the over-expressed genes under the flight condition. Genes involved in DNA repair are indicated in magenta, those coding for antioxidants in orange and identified as HGTs in light blue. Genes non-significantly differentiallyexpressed are colored in gray. X axis represents the log2foldchange and y axis a transformation of the mean expression of the genes. Fig. S7 Differential plot showing the 25 top ranked genes with highest log2foldchange values among the under-expressed genes under the flight condition. Genes involved in DNA repair are indicated in magenta, those coding for antioxidants in orange and identified as HGTs in light blue. Genes non-significantly differentiallyexpressed are colored in gray. X axis represents the log2foldchange and y axis a transformation of the mean expression of the genes. Fig. S8 Differential plot showing the genes coding for proteins involved in DNA repair over-expressed under the flight condition with highest log2foldchange values, or highest mean expression values. Genes involved in DNA repair are indicated in magenta, those coding for antioxidants in orange and identified as HGTs in light blue. Genes non-significantly differentiallyexpressed are colored in gray. X axis represents the log2foldchange and y axis a transformation of the mean expression of the genes. Fig. S9 Differential plot showing the genes coding for antioxidants over-expressed under the flight condition with highest log2foldchange values, or highest mean expression values. Genes involved in DNA repair are indicated in red, those coding for antioxidants in orange and identified as HGTs in yellow. Genes non-significantly differentiallyexpressed are colored in gray. X axis represents the log2foldchange and y axis a transformation of the mean expression of the genes. Fig. S10 Differential plot showing the genes identified as HGTs over-expressed under the flight condition with highest log2foldchange values, or highest mean expression values. Genes involved in DNA repair are indicated in magenta, those coding for antioxidants in orange and identified as HGTs in light blue. Genes non-significantly differentiallyexpressed are colored in gray. X axis represents the log2foldchange and y axis a transformation of the mean expression of the genes

Additional file 2: Movie 1. Video showcasing *A. vaga *individuals stored under conditions mirroring ground controls, including hardware. Captured immediately prior to sample fixation on December 17, 2019, the video confirms:no detectable contaminants, typical activity in hydrated bdelloids, andegg presence within autonomous cultures. Captured using Zeiss Stemi 305 Binoccular coupled with Canon camera.

Additional file 3: Tables S1-S3. Table S1 Samples sequenced and mapping statistics. The columns give the sample ID, the NCBI sample number, the GC number, the condition of the sample, its pool, the total raw reads before and after trimming, the percentage of reads after trimming, uniquely mapped, mapped to multiple or many loci and unmapped. Table S2 Genes within *Adineta vaga* genome. The columns give the gene ids from the gene assembly in Simion et al., the correspondence gene ids in the genome published by Simion et al., if the gene is annotated as HGT, the blast hits, the KEGG ids, GO ids, pfam domains, the results for all analyses with DESeq2 and EdgeR, and the TPM values for all the different analyzed conditions. The columns with the results from DESeq2 and EdgeR are explained in the second table sheet. Table S3 Temperature profiles of the two flights hardwareand the two ground controls hardware

## Data Availability

All analyses described in this study are published as supplementary tables (Additional file 3), and transcriptomic reads can be found on NCBI under the Bioproject PRJNA1142504 [55] under the biosamples SAMN42945155—SAMN42945170. Additionally, data from spaceflight are archived at HRE Data Archive (HREDA) (doi.org/10.57780/esa-yny42h0) and NASA Genelab database (identified as ROTIFER-B1). The python scripts used to calculate TPM are available on https://github.com/vmoris/TPM_script [63].

## Abbreviations

ISS: International Space Station
LEO: Low Earth Orbit
HGT: Horizontal gene transfer
DSBs: Double-strand breaks
IR: Ionizing radiation
ROS: Reactive oxygen species
ESA: European Space Agency
HR: Homologous recombination
NHEJ: Non-Homologous End-Joining
BER: Base Excision Repair
NER: Nucleotide Excision Repair
MR: Mismatch Repair
HSP: Heat shock proteins
LET: Linear Energy Transfer
DGE: Differential Gene Expression
PCA: Principal component analysis
GO: Gene Ontology
l2fc: Log2foldchange
TPM: Transcripts per million
SOD: Superoxide dismutase
FDR: False discovery rate
DDR: DNA damage response
FEP: Fluoro-ethylpolymer
CB: Culture Bag
EC: Experiment Container
EH: Experiment Hardware
