# Supplementary figures and images for: Rotifers in space: transcriptomic response of the bdelloid rotifer Adineta vaga aboard the International Space Station
Source: BMC Biol. 2025 Jul 1;23:182. doi: 10.1186/s12915-025-02272-1 (PMC12220480; doi:10.1186/s12915-025-02272-1)

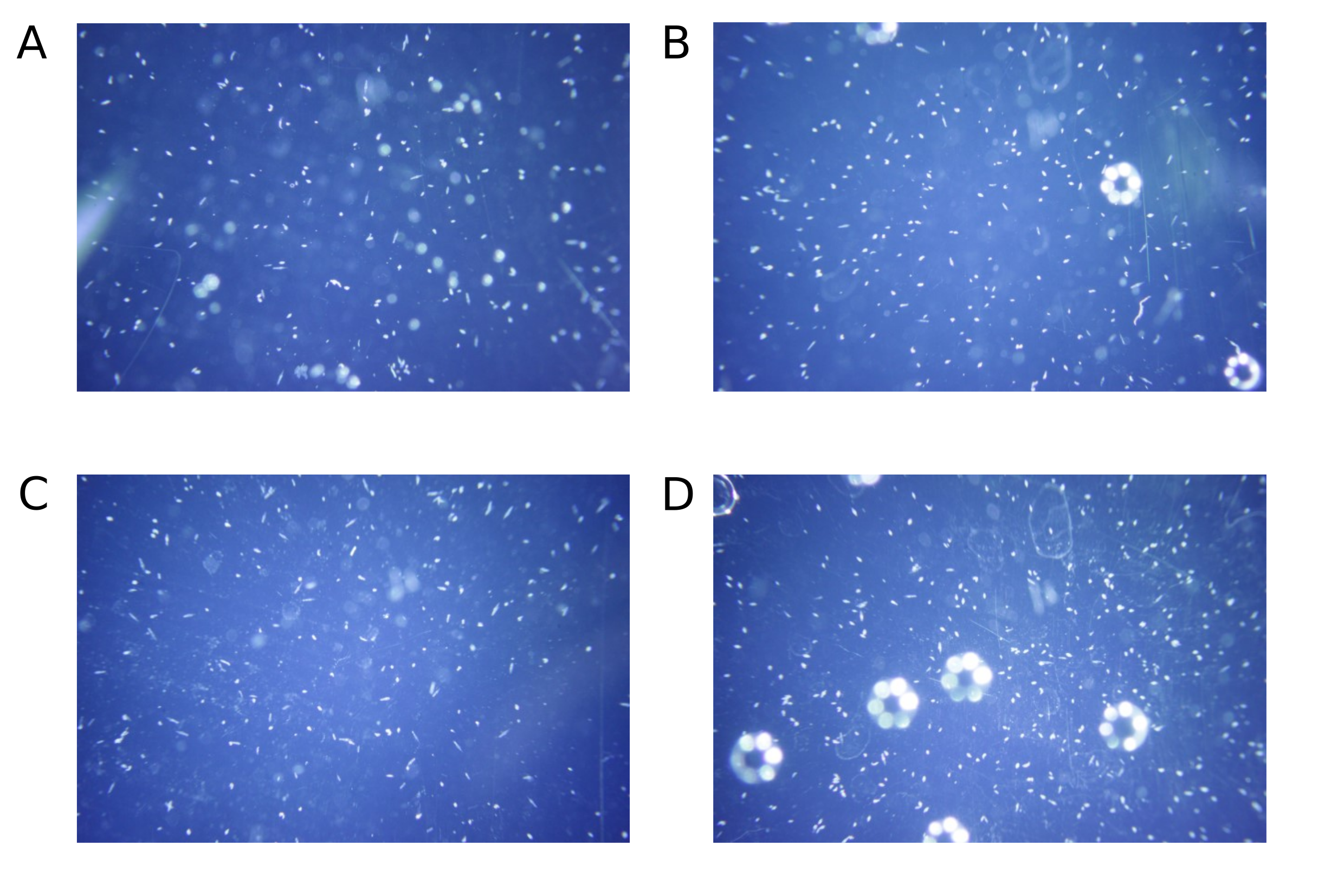

Supplement: Supplementary file 1 — Additional file 1: Figures S1-S10. Fig. S1 Overview of random A. vaga individuals loaded in PL30beforeand after leak testduring integration. Animals remain active after leak test. No modification of behavior was reported. Captured using Zeiss Stemi 305 Binoccular coupled with Canon camera. Fig. S2 Picture showcasing A. vaga individuals stored under conditions mirroring ground controls, including hardware. Captured immediately prior to sample fixation on December 17, 2019, the picture confirms:no detectable contaminants,typical activity in hydrated bdelloids, andegg presence within autonomous cultures. Captured using Zeiss Stemi 305 Binocular coupled with Canon camera. Fig. S3 Venn diagram representing the results obtained with DESeq2 and EdgeR. The genes being over-expressed with Deseq2, with EdgeR, and under-expressed with DESeq2and with EdgeR. Fig. S4 Volcano plot of genes with lowest and highest log2foldchange comparing flight and ground condition. Genes under-expressed in flight conditionare colored in dark blue while those over-expressedare colored in green. Genes differentially expressed and involved in DNA repair are colored in magenta, those coding for antioxidants in orange and identified as HGTs in light blue. Genes non-significantly differentiallyexpressed are colored in gray. Fig. S5 Frequency plots representing the number of genes with GO ids for a specific log2foldchange values identified as A) over-expressed genes or B) under-expressed genes, with a specific l2fcbeing characterizedor notwith a Gene Ontology term. Fig. S6 Differential plot showing the 25 top ranked genes with highest log2foldchange values among the over-expressed genes under the flight condition. Genes involved in DNA repair are indicated in magenta, those coding for antioxidants in orange and identified as HGTs in light blue. Genes non-significantly differentiallyexpressed are colored in gray. X axis represents the log2foldchange and y axis a transformation of the mean expression of t [file 12915_2025_2272_MOESM1_ESM.zip › Fig_S1.tiff]

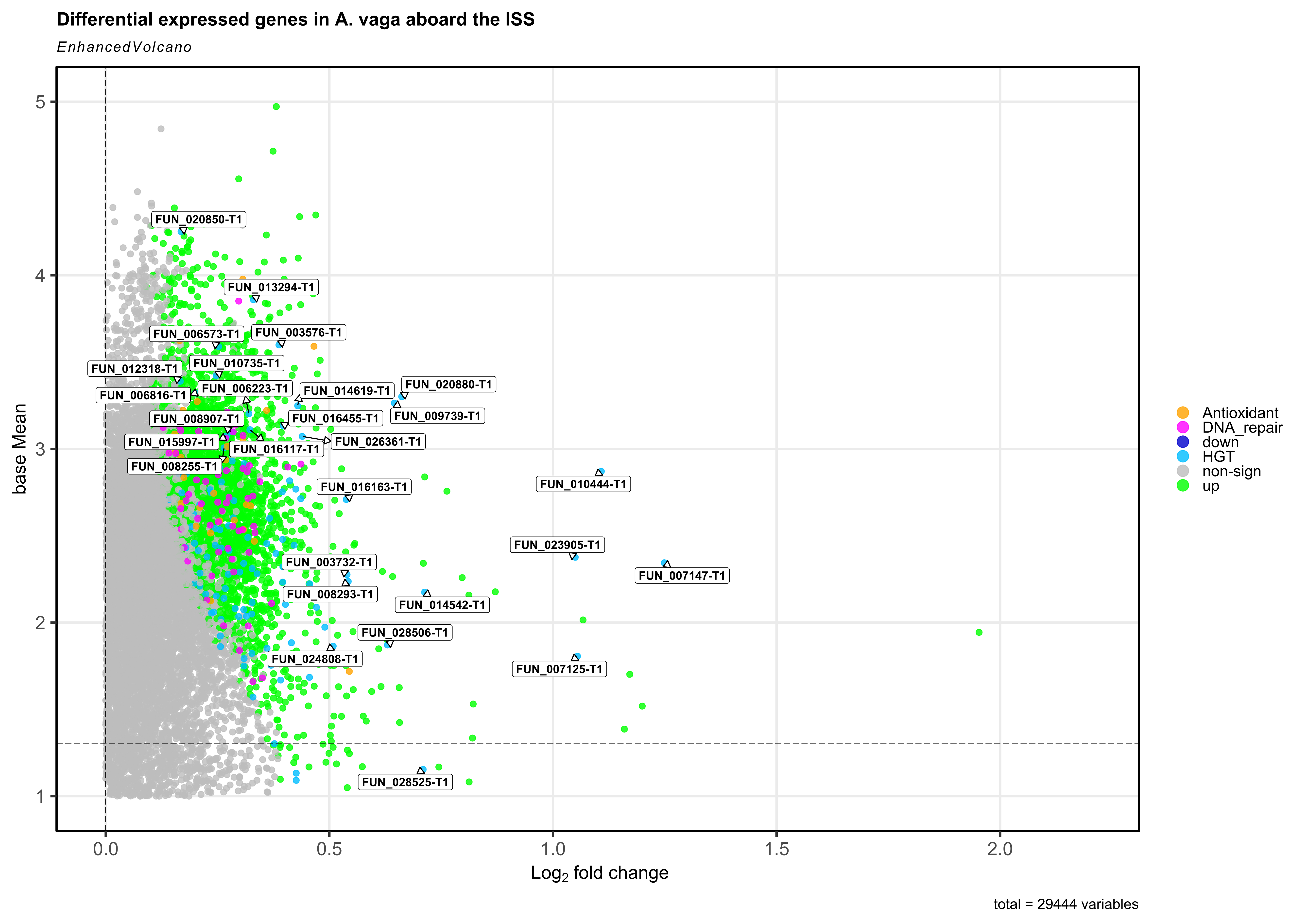

Supplement: Supplementary file 1 — Additional file 1: Figures S1-S10. Fig. S1 Overview of random A. vaga individuals loaded in PL30beforeand after leak testduring integration. Animals remain active after leak test. No modification of behavior was reported. Captured using Zeiss Stemi 305 Binoccular coupled with Canon camera. Fig. S2 Picture showcasing A. vaga individuals stored under conditions mirroring ground controls, including hardware. Captured immediately prior to sample fixation on December 17, 2019, the picture confirms:no detectable contaminants,typical activity in hydrated bdelloids, andegg presence within autonomous cultures. Captured using Zeiss Stemi 305 Binocular coupled with Canon camera. Fig. S3 Venn diagram representing the results obtained with DESeq2 and EdgeR. The genes being over-expressed with Deseq2, with EdgeR, and under-expressed with DESeq2and with EdgeR. Fig. S4 Volcano plot of genes with lowest and highest log2foldchange comparing flight and ground condition. Genes under-expressed in flight conditionare colored in dark blue while those over-expressedare colored in green. Genes differentially expressed and involved in DNA repair are colored in magenta, those coding for antioxidants in orange and identified as HGTs in light blue. Genes non-significantly differentiallyexpressed are colored in gray. Fig. S5 Frequency plots representing the number of genes with GO ids for a specific log2foldchange values identified as A) over-expressed genes or B) under-expressed genes, with a specific l2fcbeing characterizedor notwith a Gene Ontology term. Fig. S6 Differential plot showing the 25 top ranked genes with highest log2foldchange values among the over-expressed genes under the flight condition. Genes involved in DNA repair are indicated in magenta, those coding for antioxidants in orange and identified as HGTs in light blue. Genes non-significantly differentiallyexpressed are colored in gray. X axis represents the log2foldchange and y axis a transformation of the mean expression of t [file 12915_2025_2272_MOESM1_ESM.zip › Fig_S10.tiff]

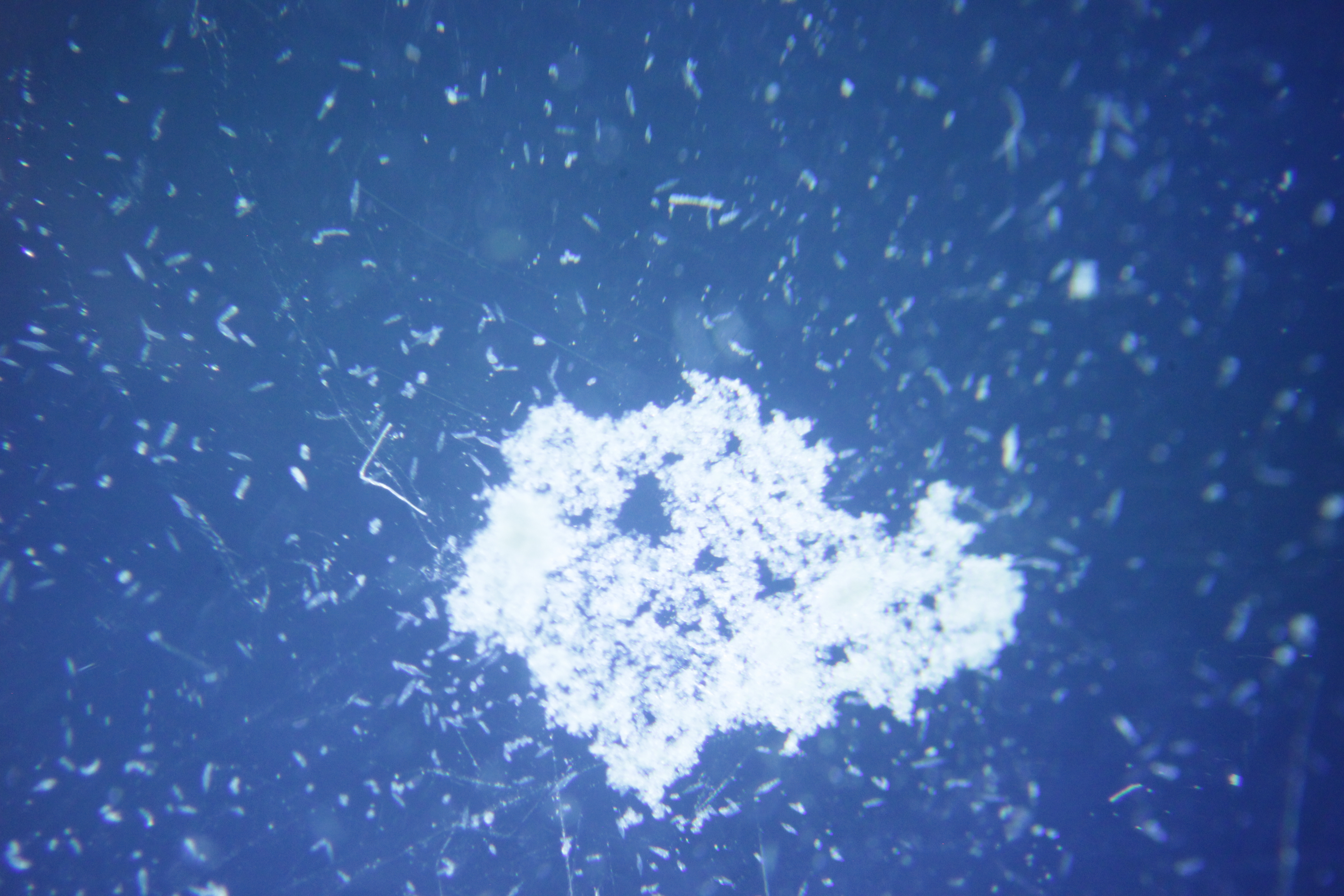

Supplement: Supplementary file 1 — Additional file 1: Figures S1-S10. Fig. S1 Overview of random A. vaga individuals loaded in PL30beforeand after leak testduring integration. Animals remain active after leak test. No modification of behavior was reported. Captured using Zeiss Stemi 305 Binoccular coupled with Canon camera. Fig. S2 Picture showcasing A. vaga individuals stored under conditions mirroring ground controls, including hardware. Captured immediately prior to sample fixation on December 17, 2019, the picture confirms:no detectable contaminants,typical activity in hydrated bdelloids, andegg presence within autonomous cultures. Captured using Zeiss Stemi 305 Binocular coupled with Canon camera. Fig. S3 Venn diagram representing the results obtained with DESeq2 and EdgeR. The genes being over-expressed with Deseq2, with EdgeR, and under-expressed with DESeq2and with EdgeR. Fig. S4 Volcano plot of genes with lowest and highest log2foldchange comparing flight and ground condition. Genes under-expressed in flight conditionare colored in dark blue while those over-expressedare colored in green. Genes differentially expressed and involved in DNA repair are colored in magenta, those coding for antioxidants in orange and identified as HGTs in light blue. Genes non-significantly differentiallyexpressed are colored in gray. Fig. S5 Frequency plots representing the number of genes with GO ids for a specific log2foldchange values identified as A) over-expressed genes or B) under-expressed genes, with a specific l2fcbeing characterizedor notwith a Gene Ontology term. Fig. S6 Differential plot showing the 25 top ranked genes with highest log2foldchange values among the over-expressed genes under the flight condition. Genes involved in DNA repair are indicated in magenta, those coding for antioxidants in orange and identified as HGTs in light blue. Genes non-significantly differentiallyexpressed are colored in gray. X axis represents the log2foldchange and y axis a transformation of the mean expression of t [file 12915_2025_2272_MOESM1_ESM.zip › Fig_S2.JPG]

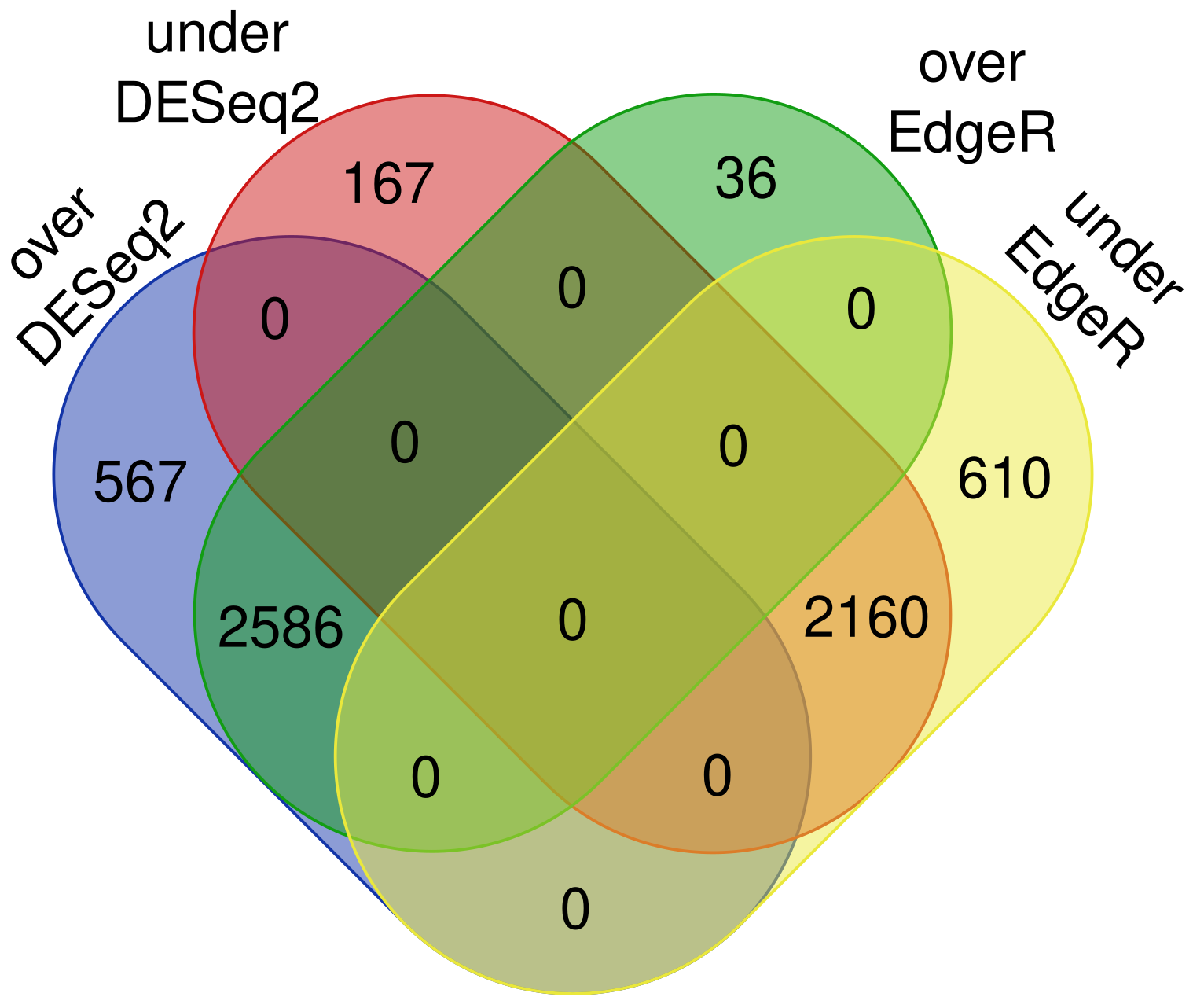

Supplement: Supplementary file 1 — Additional file 1: Figures S1-S10. Fig. S1 Overview of random A. vaga individuals loaded in PL30beforeand after leak testduring integration. Animals remain active after leak test. No modification of behavior was reported. Captured using Zeiss Stemi 305 Binoccular coupled with Canon camera. Fig. S2 Picture showcasing A. vaga individuals stored under conditions mirroring ground controls, including hardware. Captured immediately prior to sample fixation on December 17, 2019, the picture confirms:no detectable contaminants,typical activity in hydrated bdelloids, andegg presence within autonomous cultures. Captured using Zeiss Stemi 305 Binocular coupled with Canon camera. Fig. S3 Venn diagram representing the results obtained with DESeq2 and EdgeR. The genes being over-expressed with Deseq2, with EdgeR, and under-expressed with DESeq2and with EdgeR. Fig. S4 Volcano plot of genes with lowest and highest log2foldchange comparing flight and ground condition. Genes under-expressed in flight conditionare colored in dark blue while those over-expressedare colored in green. Genes differentially expressed and involved in DNA repair are colored in magenta, those coding for antioxidants in orange and identified as HGTs in light blue. Genes non-significantly differentiallyexpressed are colored in gray. Fig. S5 Frequency plots representing the number of genes with GO ids for a specific log2foldchange values identified as A) over-expressed genes or B) under-expressed genes, with a specific l2fcbeing characterizedor notwith a Gene Ontology term. Fig. S6 Differential plot showing the 25 top ranked genes with highest log2foldchange values among the over-expressed genes under the flight condition. Genes involved in DNA repair are indicated in magenta, those coding for antioxidants in orange and identified as HGTs in light blue. Genes non-significantly differentiallyexpressed are colored in gray. X axis represents the log2foldchange and y axis a transformation of the mean expression of t [file 12915_2025_2272_MOESM1_ESM.zip › Fig_S3.tiff]

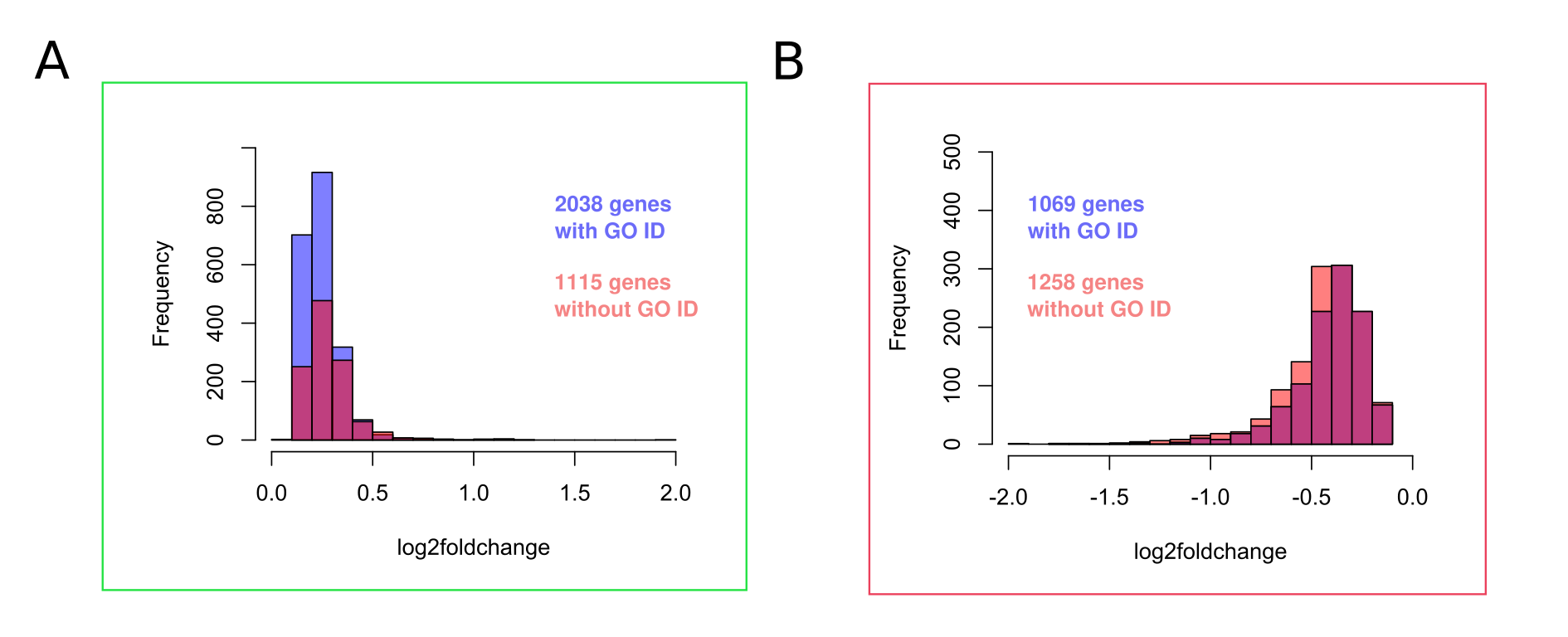

Supplement: Supplementary file 1 — Additional file 1: Figures S1-S10. Fig. S1 Overview of random A. vaga individuals loaded in PL30beforeand after leak testduring integration. Animals remain active after leak test. No modification of behavior was reported. Captured using Zeiss Stemi 305 Binoccular coupled with Canon camera. Fig. S2 Picture showcasing A. vaga individuals stored under conditions mirroring ground controls, including hardware. Captured immediately prior to sample fixation on December 17, 2019, the picture confirms:no detectable contaminants,typical activity in hydrated bdelloids, andegg presence within autonomous cultures. Captured using Zeiss Stemi 305 Binocular coupled with Canon camera. Fig. S3 Venn diagram representing the results obtained with DESeq2 and EdgeR. The genes being over-expressed with Deseq2, with EdgeR, and under-expressed with DESeq2and with EdgeR. Fig. S4 Volcano plot of genes with lowest and highest log2foldchange comparing flight and ground condition. Genes under-expressed in flight conditionare colored in dark blue while those over-expressedare colored in green. Genes differentially expressed and involved in DNA repair are colored in magenta, those coding for antioxidants in orange and identified as HGTs in light blue. Genes non-significantly differentiallyexpressed are colored in gray. Fig. S5 Frequency plots representing the number of genes with GO ids for a specific log2foldchange values identified as A) over-expressed genes or B) under-expressed genes, with a specific l2fcbeing characterizedor notwith a Gene Ontology term. Fig. S6 Differential plot showing the 25 top ranked genes with highest log2foldchange values among the over-expressed genes under the flight condition. Genes involved in DNA repair are indicated in magenta, those coding for antioxidants in orange and identified as HGTs in light blue. Genes non-significantly differentiallyexpressed are colored in gray. X axis represents the log2foldchange and y axis a transformation of the mean expression of t [file 12915_2025_2272_MOESM1_ESM.zip › Fig_S5.tiff]

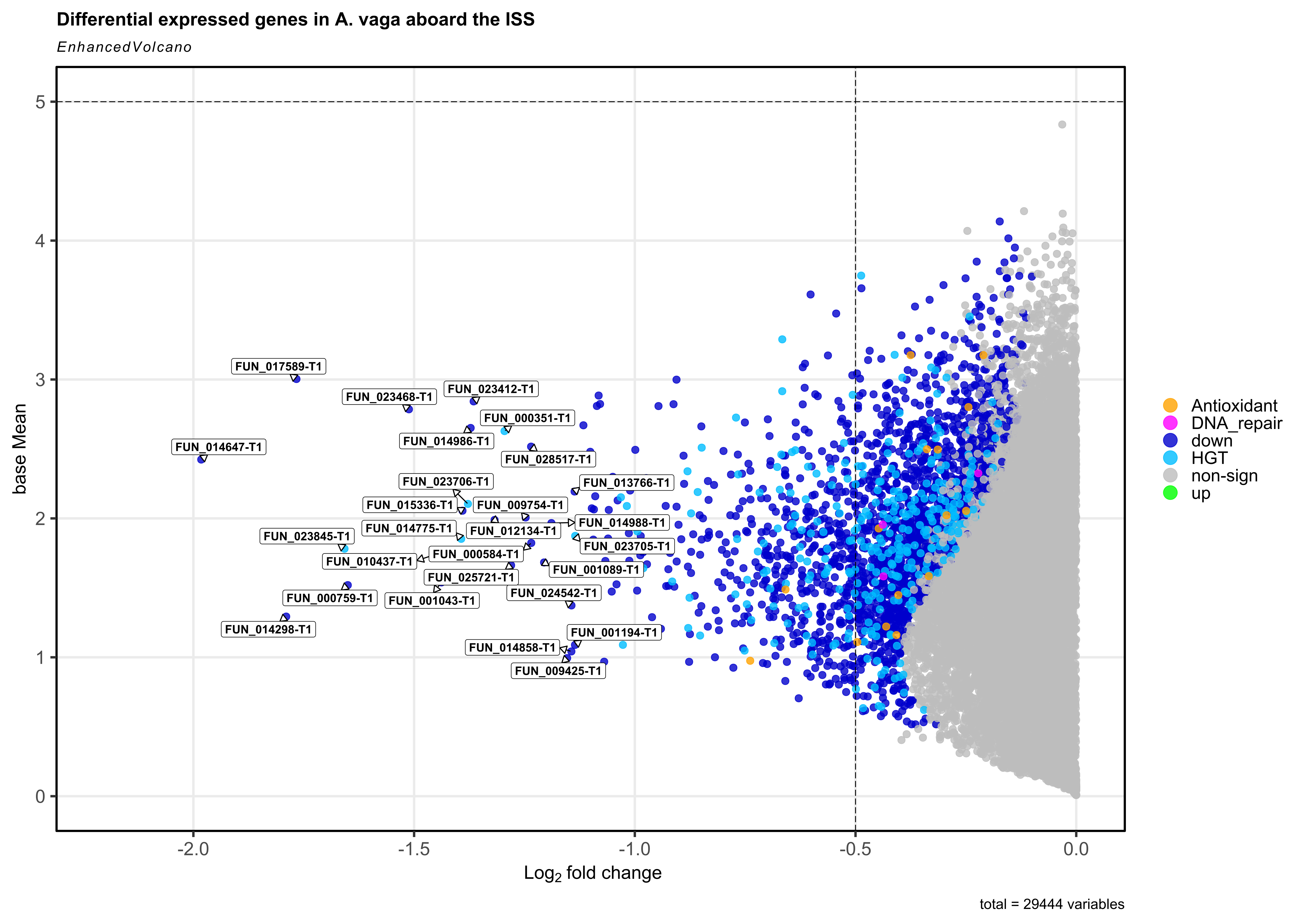

Supplement: Supplementary file 1 — Additional file 1: Figures S1-S10. Fig. S1 Overview of random A. vaga individuals loaded in PL30beforeand after leak testduring integration. Animals remain active after leak test. No modification of behavior was reported. Captured using Zeiss Stemi 305 Binoccular coupled with Canon camera. Fig. S2 Picture showcasing A. vaga individuals stored under conditions mirroring ground controls, including hardware. Captured immediately prior to sample fixation on December 17, 2019, the picture confirms:no detectable contaminants,typical activity in hydrated bdelloids, andegg presence within autonomous cultures. Captured using Zeiss Stemi 305 Binocular coupled with Canon camera. Fig. S3 Venn diagram representing the results obtained with DESeq2 and EdgeR. The genes being over-expressed with Deseq2, with EdgeR, and under-expressed with DESeq2and with EdgeR. Fig. S4 Volcano plot of genes with lowest and highest log2foldchange comparing flight and ground condition. Genes under-expressed in flight conditionare colored in dark blue while those over-expressedare colored in green. Genes differentially expressed and involved in DNA repair are colored in magenta, those coding for antioxidants in orange and identified as HGTs in light blue. Genes non-significantly differentiallyexpressed are colored in gray. Fig. S5 Frequency plots representing the number of genes with GO ids for a specific log2foldchange values identified as A) over-expressed genes or B) under-expressed genes, with a specific l2fcbeing characterizedor notwith a Gene Ontology term. Fig. S6 Differential plot showing the 25 top ranked genes with highest log2foldchange values among the over-expressed genes under the flight condition. Genes involved in DNA repair are indicated in magenta, those coding for antioxidants in orange and identified as HGTs in light blue. Genes non-significantly differentiallyexpressed are colored in gray. X axis represents the log2foldchange and y axis a transformation of the mean expression of t [file 12915_2025_2272_MOESM1_ESM.zip › Fig_S7.tiff]

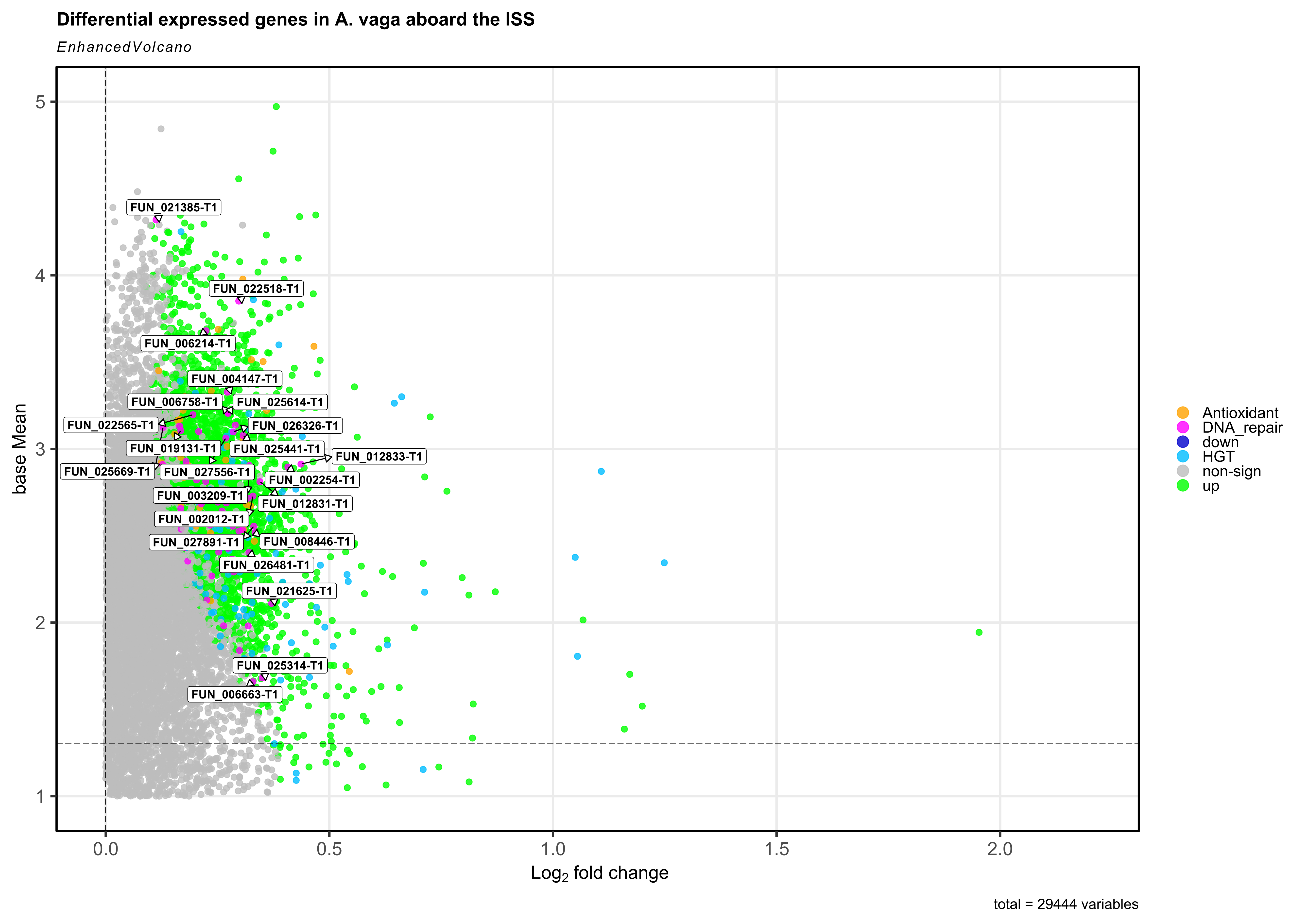

Supplement: Supplementary file 1 — Additional file 1: Figures S1-S10. Fig. S1 Overview of random A. vaga individuals loaded in PL30beforeand after leak testduring integration. Animals remain active after leak test. No modification of behavior was reported. Captured using Zeiss Stemi 305 Binoccular coupled with Canon camera. Fig. S2 Picture showcasing A. vaga individuals stored under conditions mirroring ground controls, including hardware. Captured immediately prior to sample fixation on December 17, 2019, the picture confirms:no detectable contaminants,typical activity in hydrated bdelloids, andegg presence within autonomous cultures. Captured using Zeiss Stemi 305 Binocular coupled with Canon camera. Fig. S3 Venn diagram representing the results obtained with DESeq2 and EdgeR. The genes being over-expressed with Deseq2, with EdgeR, and under-expressed with DESeq2and with EdgeR. Fig. S4 Volcano plot of genes with lowest and highest log2foldchange comparing flight and ground condition. Genes under-expressed in flight conditionare colored in dark blue while those over-expressedare colored in green. Genes differentially expressed and involved in DNA repair are colored in magenta, those coding for antioxidants in orange and identified as HGTs in light blue. Genes non-significantly differentiallyexpressed are colored in gray. Fig. S5 Frequency plots representing the number of genes with GO ids for a specific log2foldchange values identified as A) over-expressed genes or B) under-expressed genes, with a specific l2fcbeing characterizedor notwith a Gene Ontology term. Fig. S6 Differential plot showing the 25 top ranked genes with highest log2foldchange values among the over-expressed genes under the flight condition. Genes involved in DNA repair are indicated in magenta, those coding for antioxidants in orange and identified as HGTs in light blue. Genes non-significantly differentiallyexpressed are colored in gray. X axis represents the log2foldchange and y axis a transformation of the mean expression of t [file 12915_2025_2272_MOESM1_ESM.zip › Fig_S8.tiff]

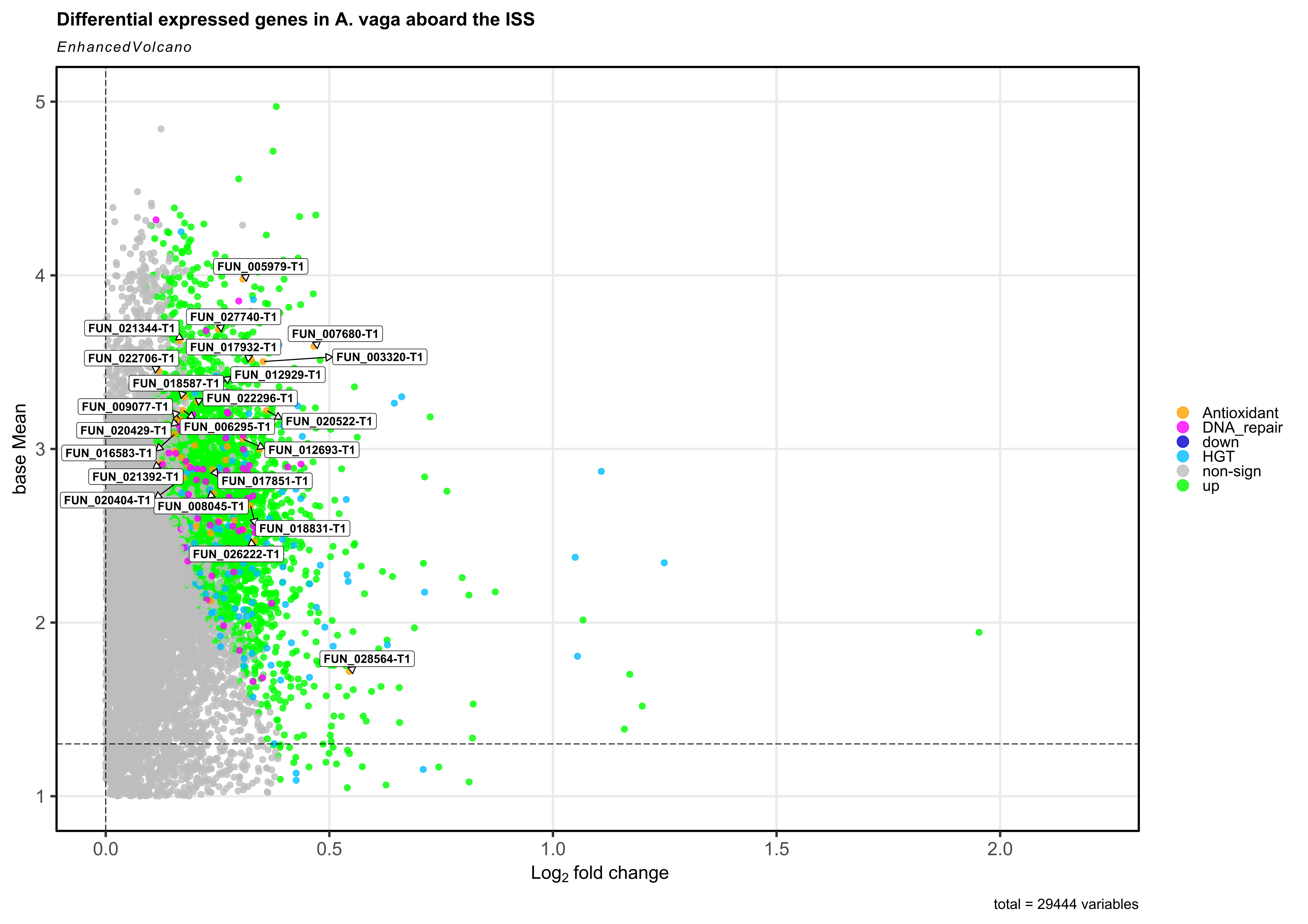

Supplement: Supplementary file 1 — Additional file 1: Figures S1-S10. Fig. S1 Overview of random A. vaga individuals loaded in PL30beforeand after leak testduring integration. Animals remain active after leak test. No modification of behavior was reported. Captured using Zeiss Stemi 305 Binoccular coupled with Canon camera. Fig. S2 Picture showcasing A. vaga individuals stored under conditions mirroring ground controls, including hardware. Captured immediately prior to sample fixation on December 17, 2019, the picture confirms:no detectable contaminants,typical activity in hydrated bdelloids, andegg presence within autonomous cultures. Captured using Zeiss Stemi 305 Binocular coupled with Canon camera. Fig. S3 Venn diagram representing the results obtained with DESeq2 and EdgeR. The genes being over-expressed with Deseq2, with EdgeR, and under-expressed with DESeq2and with EdgeR. Fig. S4 Volcano plot of genes with lowest and highest log2foldchange comparing flight and ground condition. Genes under-expressed in flight conditionare colored in dark blue while those over-expressedare colored in green. Genes differentially expressed and involved in DNA repair are colored in magenta, those coding for antioxidants in orange and identified as HGTs in light blue. Genes non-significantly differentiallyexpressed are colored in gray. Fig. S5 Frequency plots representing the number of genes with GO ids for a specific log2foldchange values identified as A) over-expressed genes or B) under-expressed genes, with a specific l2fcbeing characterizedor notwith a Gene Ontology term. Fig. S6 Differential plot showing the 25 top ranked genes with highest log2foldchange values among the over-expressed genes under the flight condition. Genes involved in DNA repair are indicated in magenta, those coding for antioxidants in orange and identified as HGTs in light blue. Genes non-significantly differentiallyexpressed are colored in gray. X axis represents the log2foldchange and y axis a transformation of the mean expression of t [file 12915_2025_2272_MOESM1_ESM.zip › Fig_S9.tiff]
