# Supplementary material for: Rotifers in space: transcriptomic response of the bdelloid rotifer Adineta vaga aboard the International Space Station
Source: BMC Biol. 2025 Jul 1;23:182. doi: 10.1186/s12915-025-02272-1 (PMC12220480; doi:10.1186/s12915-025-02272-1)
Supplement: Supplementary file 3 — Additional file 3: Tables S1-S3. Table S1 Samples sequenced and mapping statistics. The columns give the sample ID, the NCBI sample number, the GC number, the condition of the sample, its pool, the total raw reads before and after trimming, the percentage of reads after trimming, uniquely mapped, mapped to multiple or many loci and unmapped. Table S2 Genes within Adineta vaga genome. The columns give the gene ids from the gene assembly in Simion et al., the correspondence gene ids in the genome published by Simion et al., if the gene is annotated as HGT, the blast hits, the KEGG ids, GO ids, pfam domains, the results for all analyses with DESeq2 and EdgeR, and the TPM values for all the different analyzed conditions. The columns with the results from DESeq2 and EdgeR are explained in the second table sheet. Table S3 Temperature profiles of the two flights hardwareand the two ground controls hardware [file 12915_2025_2272_MOESM3_ESM.zip › Table_S1_Samples sequenced and mapping statistics.docx]

**Table S1** **Samples sequenced and mapping statistics**. The columns give the sample ID, the NCBI sample number, the GC number, the condition of the sample, its pool, the total raw reads before and after trimming, the percentage of reads after trimming, uniquely mapped, mapped to multiple or many loci and unmapped.

| **Sample** | **NCBI sample Number** | **GC Number** | **Condition** | **Pool** | **Total raw reads** | **Total reads after trimming** | **Percentage after trimming (%)** | **uniquely mapped (%)** | **mapped to multiple loci (%)** | **mapped to too many loci (%)** | **Unmapped (%)** |
| --- | --- | --- | --- | --- | --- | --- | --- | --- | --- | --- | --- |
| flight 1 | SAMN42945155 | GC1002250 | Flight | Pool 1 | 11060121 | 10958044 | 99.08 | 86.93 | 4.8 | 0.1 | 8.18 |
| flight 9 | SAMN42945156 | GC1002253 | Flight | Pool 1 | 9944125 | 9854942 | 99.10 | 87.21 | 5.21 | 0.11 | 7.46 |
| flight 3 | SAMN42945157 | GC1002725 | Flight | Pool 1 | 23006783 | 22932660 | 99.68 | 88.74 | 5.03 | 0.13 | 6.11 |
| flight 7 | SAMN42945158 | GC1002726 | Flight | Pool 1 | 22543412 | 22370442 | 99.23 | 89.11 | 4.75 | 0.15 | 5.99 |
| flight 4 | SAMN42945159 | GC1002251 | Flight | Pool 2 | 8930588 | 8836485 | 98.95 | 86.59 | 4.34 | 0.17 | 8.9 |
| flight 6 | SAMN42945160 | GC1002252 | Flight | Pool 2 | 9729859 | 9615672 | 98.83 | 87.05 | 4.89 | 0.09 | 7.97 |
| flight 2 | SAMN42945161 | GC1002724 | Flight | Pool 2 | 16559696 | 16468719 | 99.45 | 87.67 | 4.77 | 0.14 | 7.43 |
| flight 8 | SAMN42945162 | GC1002727 | Flight | Pool 2 | 22335954 | 22236562 | 99.56 | 88.22 | 4.93 | 0.16 | 6.7 |
| ground 13 | SAMN42945163 | GC1002256 | Ground | Pool 1 | 9002879 | 8890551 | 98.75 | 85.67 | 5.01 | 0.14 | 9.19 |
| ground 21 | SAMN42945164 | GC1002257 | Ground | Pool 1 | 10992966 | 10878506 | 98.96 | 85.83 | 4.98 | 1.01 | 8.18 |
| ground 15 | SAMN42945165 | GC1002729 | Ground | Pool 1 | 24369529 | 24259530 | 99.55 | 88.85 | 4.8 | 0.13 | 6.22 |
| ground 19 | SAMN42945166 | GC1002730 | Ground | Pool 1 | 23575585 | 23461484 | 99.52 | 88.07 | 4.76 | 0.16 | 7 |
| ground 16 | SAMN42945167 | GC1002254 | Ground | Pool 2 | 11064447 | 10953619 | 99.00 | 86.44 | 4.73 | 0.14 | 8.69 |
| ground 18 | SAMN42945168 | GC1002255 | Ground | Pool 2 | 9542544 | 9412860 | 98.64 | 85.68 | 4.79 | 0.12 | 9.41 |
| ground 14 | SAMN42945169 | GC1002728 | Ground | Pool 2 | 25397891 | 25217031 | 99.29 | 87.83 | 4.56 | 0.18 | 7.44 |
| ground 20 | SAMN42945170 | GC1002731 | Ground | Pool 2 | 44406852 | 44234562 | 99.61 | 87.42 | 4.79 | 1.68 | 6.12 |
